# Computational prioritization of candidate TCDD-associated targets in retinoblastoma using network toxicology and transcriptomic analysis

**DOI:** 10.3389/fgene.2026.1911449

**Published:** 2026-09-09

**Authors:** Yingluan Wang, Qiang Hu, Zhan Cao, Dian Ye, Dawei Sun, Lijun Qu

**Affiliations:** 1 Department of Ophthalmology, The Second Affiliated Hospital of Harbin Medical University, Harbin, China; 2 Department of Neurology, The Second Affiliated Hospital of Harbin Medical University, Harbin, China

**Keywords:** Cdk1, environmental exposure, machine learning, molecular docking, network toxicology, retinoblastoma, TCDD

## Abstract

**Background:**

Retinoblastoma (RB) is the most common ocular malignancy in children, but the relationship between environmental pollutants and RB-related molecular alterations remains unclear. This study aimed to identify candidate targets and pathways potentially linking 2.3,7,8-tetrachlorodibenzo-p-dioxin (TCDD) with RB.

**Methods:**

Network toxicology, computational toxicology, and integrated RB transcriptomic analyses were used to identify candidate TCDD-RB targets. WGCNA, eight machine-learning algorithms, and SHAP analysis were applied to screen hub genes and evaluate their contributions to model prediction. Functional enrichment analysis, GSEA/GSVA, immune microenvironment analysis, molecular docking, and expression validation in RB cell lines were subsequently performed to assess the potential biological relevance of the hub genes.

**Results:**

A total of 18 candidate TCDD-RB targets were identified and were mainly enriched in cell-cycle regulation, G2/M transition, DNA replication, and the p53 signaling pathway. Machine learning further identified five hub genes, namely, CA14, PIP5K1B, GRIA2, KDR, and CDK1. Among them, CDK1 was significantly upregulated in RB and was associated with proliferation-related pathways, including the cell cycle, DNA replication, and mismatch repair. The other four genes were significantly downregulated and may be involved in metabolic homeostasis and immune microenvironment regulation. Molecular docking predicted potential structural compatibility between TCDD and all five hub proteins, with CDK1 showing the most favorable predicted docking energy. qRT-PCR analysis in RB cell lines supported the differential expression patterns of the five hub genes, and qRT-PCR and Western blotting confirmed the efficiency of CDK1 overexpression and knockdown.

**Conclusion:**

This study delineated a candidate molecular network at the intersection of TCDD-associated toxic This study delineated a candidate molecular network at the intersection of TCDD-associated toxicological targets and RB-related transcriptomic alterations. CDK1 emerged as a candidate hub associated with cell-cycle dysregulation, whereas CA14, PIP5K1B, GRIA2, and KDR were linked to broader disturbances in cellular homeostasis and microenvironmental regulation. Because these findings are based primarily on computational analyses and preliminary expression validation, they should be regarded as hypothesis-generating. Direct TCDD exposure and functional studies are required to determine whether TCDD functionally affects these genes or RB-related cellular phenotypes.

## 1 Introduction

Retinoblastoma (RB) is the most common primary intraocular malignant tumor in children, accounting for approximately 4% of paediatric malignancies, with about 8,000 new cases diagnosed worldwide each year. The development of RB is closely associated with inactivation of the RB1 gene, and approximately 40% of cases have a hereditary background (Vaithianathan and Zanazzi, 2026). Although advances in early screening, ophthalmic imaging, and comprehensive treatment strategies have increased the survival rate of children with RB in high-income countries to nearly 100%, substantial global disparities remain in disease burden and clinical outcomes. Previous studies have shown that, with relatively well-established screening and healthcare systems, the median age at diagnosis in high-income countries is approximately 14.1 months; in contrast, the median age at diagnosis in low-income countries may be delayed to 30.5 months, with survival rates decreasing to around 50% (Wang et al., 2025). Countries and regions with large populations, high birth rates, or relatively limited medical resources, such as India, China, and parts of Africa, bear a considerable disease burden (Kumari et al., 2025). Inadequate screening systems, limited public awareness, and delayed diagnosis remain important factors contributing to an increased risk of extraocular invasion, distant metastasis, and death among children with RB in low-income regions.

In addition to genetic alterations and disparities in diagnostic and treatment resources, the potential contribution of environmental exposures to RB development has received increasing attention. Children undergo rapid growth and development and may therefore be particularly susceptible to exogenous pollutants. Persistent environmental pollutants can affect child health by disrupting endocrine homeostasis, neurodevelopment, immune regulation, and cell proliferation. A study based on neonatal dried blood spots reported that higher levels of perfluorooctane sulfonate and perfluorooctanoic acid were associated with an increased risk of childhood RB (Chen et al., 2024). Perinatal exposure to benzene, chloroform, chromium, nickel, and several traffic- and industry-related air toxicants has also been associated with an increased risk of RB (Heck et al., 2015). A recent review of ophthalmotoxicity further suggested that persistent polyhalogenated compounds, including PFAS, PCBs, PBDEs, PCDDs, and PCDFs, may affect the ocular system and may be associated with retinal degeneration, cataracts, and RB; however, the molecular basis of their ocular toxicity remains insufficiently understood (Louzon et al., 2026). Current evidence has largely focused on epidemiological associations between environmental exposures and RB risk, whereas the molecular targets of specific pollutants and their relationships with RB-associated molecular alterations have not been systematically investigated.

2.3,7,8-Tetrachlorodibenzo-p-dioxin (TCDD) is a typical persistent organic pollutant mainly generated from industrial processes such as waste incineration, metal smelting, and the production of chlorinated chemicals. TCDD exhibits strong environmental persistence, bioaccumulation, and systemic toxicity, and its classical toxic effects are mainly mediated through the aryl hydrocarbon receptor (AHR) pathway. After binding to AHR, TCDD can regulate the expression of multiple downstream genes, thereby affecting xenobiotic metabolism, oxidative stress, immune regulation, endocrine homeostasis, and tumor-related signaling pathways.

Previous studies have suggested that, beyond its well-characterized AHR-mediated toxic effects, TCDD can influence ocular tissues and cell-cycle-related processes. Takeuchi et al. showed that TCDD induced CYP1A1 and VEGF-A expression in the mouse retina and retinal pigment epithelium and exacerbated experimental choroidal neovascularization, indicating that TCDD can affect ocular angiogenic processes through AHR-related mechanisms (Takeuchi et al., 2009). These findings provide direct experimental evidence that TCDD can induce biological responses in ocular tissues, although its effects on RB were not examined.

TCDD-AHR signaling has also been implicated in cell-cycle regulation. Huang and Elferink demonstrated that TCDD-induced AHR-mediated G1 arrest was dependent on ARNT and involved functional interactions among AHR, hypophosphorylated pRb, and the E2F regulatory system (Huang and Elferink, 2005). Faust et al. further showed that TCDD suppressed the G1-to-S-phase transition in an AHR-dependent manner, accompanied by decreased cyclin D1 and cyclin A expression, increased p27 expression, and reduced pRb phosphorylation (Faust et al., 2013). Although these studies were not performed in RB models, they indicate that TCDD-AHR signaling can affect pRb/E2F- and cyclin-CDK-related cell-cycle processes, providing a biological rationale for investigating the relationship between TCDD-associated toxicological effects and cell-cycle dysregulation in RB.

However, the relationship between TCDD-associated toxicological effects and RB-related molecular alterations has not been systematically investigated. It remains unclear which TCDD-associated targets are aberrantly expressed in RB, whether they participate in RB-related co-expression modules, and which biological pathways and regulatory processes they primarily affect. Systematic transcriptomic and network-level evidence linking TCDD-associated molecular targets to cell-cycle dysregulation, abnormalities in DNA replication and repair, and alterations in the tumor microenvironment in RB remains lacking.

**FIGURE 1:**
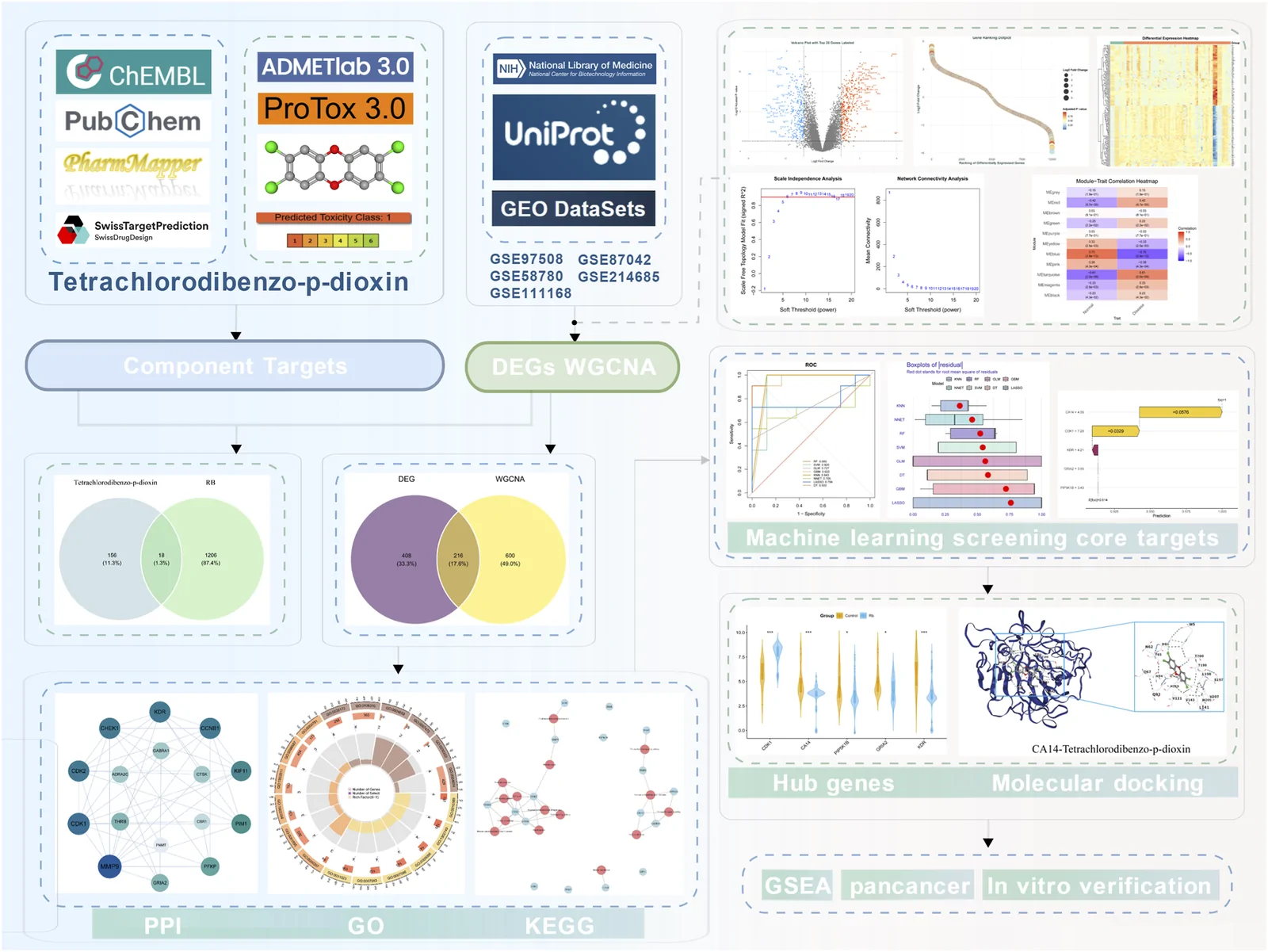
Flowchart of the study.

To address these questions, the present study integrated network toxicology, computational toxicology, RB transcriptomic analysis, weighted gene co-expression network analysis, machine learning, interpretable modelling, functional enrichment analysis, molecular docking, and *in vitro* expression validation (Mahmoudi et al., 2024; Wang et al., 2024). We systematically identified candidate genes supported by both TCDD-associated target predictions and RB transcriptomic alterations and characterized their associated biological pathways and network regulatory features. Molecular docking was used to evaluate the predicted structural compatibility between TCDD and the candidate proteins, whereas experiments in RB cell lines were performed to assess the expression patterns of the candidate genes and confirm the efficiency of CDK1 overexpression and knockdown. This study provides an integrative framework for characterizing the potential molecular relationships between TCDD-associated toxicological targets and RB-related molecular alterations and prioritizes candidates for subsequent direct TCDD exposure experiments, AHR pathway intervention, functional validation, and *in vivo* investigation. Figure 1 shows the overall workflow of this study.

## 2 Materials and methods

### 2.1 Acquisition of chemical components and targets of TCDD

To systematically analyze the molecular features and predicted target profile of TCDD, this study used a multi-source database integration strategy for feature analysis. The chemical structure of TCDD and its simplified molecular-input line-entry system (SMILES) representation were obtained from the PubChem database (https://pubchem.ncbi.nlm.nih.gov/) (Kim et al., 2023). Potential targets of TCDD were predicted using ChEMBL (https://www.ebi.ac.uk/chembl/) (Mendez et al., 2019), Swiss Target Prediction (http://swisstargetprediction.ch/) (Daina et al., 2019), and PharmMapper (http://www.lilab-ecust.cn/pharmmapper/) (Chen et al., 2026), with “*Homo sapiens*” set as the species, so that the potential action targets of TCDD could be systematically screened. All predicted targets were then cross-validated and standardized using the UniProt database to remove identifier differences from different sources and ensure data consistency (Liu et al., 2026). After removing duplicate entries and integrating all targets, a unified database of potential TCDD targets was constructed for subsequent analysis.

### 2.2 Computational toxicity profiling of TCDD

To comprehensively analyze the toxicological features of TCDD, this study carried out an initial assessment by combining computational prediction tools with a literature review. ADMETlab 3.0 (https://admetlab3.scbdd.com/server/evaluationCal) (Xiong et al., 2021) and ProTox 3.0 (https://tox.charite.de/protox3/) were used as computational toxicology platforms to obtain toxicity profiles (Banerjee et al., 2024), absorption, distribution, metabolism, excretion, and toxicity (ADMET) properties, and potential risk indicators through online prediction algorithms. All predicted results were then cross-checked with published literature data, and a comprehensive toxicological profile of TCDD was constructed.

### 2.3 Acquisition of RB-related targets

In this study, five RB transcriptomic datasets (GSE97508, GSE58780, GSE111168, GSE87042, and GSE214685) were selected from the National Center for Biotechnology Information Gene Expression Omnibus (NCBI GEO) (https://www.ncbi.nlm.nih.gov/geo/) database (Guan et al., 2025; Kang and Liu, 2026; Liu H et al., 2025). These datasets were selected because they contained human RB-related samples and corresponding controls, with accessible expression matrices and clearly defined sample groups. Their sample sizes and available clinical or sample characteristics are summarized in Supplementary Table S2. Potential heterogeneity among the datasets was mainly related to differences in sample source, control type, and experimental platform. To establish a robust analytical framework, GSE97508, GSE58780, and GSE111168 were designated as the discovery cohort, while GSE87042 and GSE214685 served as independent validation cohorts. Before integration, each discovery dataset was log2-transformed where necessary and normalized separately using platform-appropriate procedures. Unannotated expression features were removed, features mapped to the same gene were averaged, and features below the dataset-specific first-quartile expression threshold in more than 50% of samples were excluded. Samples outside the 95% Principal Component Analysis (PCA) confidence ellipse of their biological group and consistently separated from other samples within the same group by unsupervised hierarchical clustering were excluded as outliers. Only shared genes were retained for integration. Batch effects were then corrected using the ComBat algorithm implemented in the sva package (v3.56.0) under a parametric empirical Bayesian framework (Leek et al., 2012). The effectiveness of batch-effect correction and data integration was evaluated by comparing PCA results before and after ComBat correction.

### 2.4 Differential gene expression analysis

Differentially expressed genes (DEGs) were identified using the limma package. The screening criteria were set as a false discovery rate (FDR)-adjusted *P* value <0.05 and an absolute log2 fold change (log2FC) > 1, which indicates a two-fold change in expression. All significant differential expression results were visualized using the ggplot2 package (Li et al., 2026).

### 2.5 Weighted gene co-expression network analysis (WGCNA)

To construct a WGCNA, this study used the WGCNA R package for systematic analysis (Liu X et al., 2025). Sample clustering was first performed for quality control, and outlier samples were removed. Then, the optimal soft-thresholding power was selected to meet the scale-free network criterion (*R* ^2^ > 0.85). Module identification was performed based on the topological overlap matrix (TOM), with the minimum module size set to 30 and the merge cut height set to 0.25. Biologically relevant modules were screened by calculating Pearson correlation coefficients between module eigengenes and phenotypes (|R| > 0.5, *P* < 0.05). Finally, hub genes were identified from key modules based on intramodular connectivity (kME >0.8).

### 2.6 Identification of candidate TCDD-RB overlapping targets

To identify candidate overlapping targets linking predicted TCDD-associated targets with RB-related molecular alterations, we combined WGCNA-identified RB-related module genes with RB-associated DEGs, removed duplicates to obtain a unified set of RB-associated core genes, intersected this set with TCDD-predicted targets, and visualized the overlapping genes as TCDD-RB core targets using a Venn diagram.

### 2.7 PPI network analysis

To further analyze the interactions among TCDD-RB key targets, this study constructed a protein-protein interaction (PPI) network (Szklarczyk et al., 2023). The gene list was submitted to the STRING database (v12.0, https://string-db.org), with “*H. sapiens*” set as the species. The obtained interaction data were then imported into Cytoscape software (v3.10.1) for network visualization and topological analysis, so that the functional roles of key proteins in the network could be explored (Zheng et al., 2025).

### 2.8 Functional enrichment analysis

Functional enrichment analysis was performed using the clusterProfiler package (v4.12.2) (Yang et al., 2026). Gene Ontology (GO) enrichment analysis (Pitarch et al., 2025) and Kyoto Encyclopedia of Genes and Genomes (KEGG) pathway enrichment analysis (Kanehisa et al., 2022)were carried out separately. GO analysis included three categories: biological process, cellular component, and molecular function. An FDR-adjusted *P* value <0.05 was set as the significance threshold to identify functions and pathways that were significantly enriched in TCDD-related RB (Si et al., 2026).

### 2.9 Machine learning-based core gene screening

This study used an integrated machine-learning approach to evaluate 18 candidate genes. Based on the integrated GSE97508, GSE58780, and GSE111168 datasets, independent training and validation datasets were established. Eight machine-learning models were constructed, including random forest (RF), support vector machine (SVM), generalized linear model (GLM), gradient boosting machine (GBM), k-nearest neighbors (KNN), neural network (NNET), penalized logistic regression based on glmnet, and decision tree (DT). Model training and internal optimization were performed using five-fold cross-validation. Model performance was evaluated in the validation dataset by residual analysis and receiver operating characteristic analysis, with the area under the curve (AUC) used to assess discriminative performance. Permutation-based feature importance was calculated separately for each model using root mean squared error as the loss function. Correlations among the selected genes were evaluated using Pearson correlation analysis and visualized using the circlize and GGally packages. The diagnostic performance of each gene was assessed using ROC curves generated with the pROC package. Differences in gene expression between the control and RB groups were evaluated using the Wilcoxon rank-sum test, with *P* values adjusted using the Benjamini–Hochberg method. Violin plots were generated using the ggplot2 and ggpubr packages.

### 2.10 Shapley additive explanations (SHAP)-based model interpretability analysis

SHAP analysis was used to evaluate the contribution of each feature to model prediction, without implying biological causality. Based on the integrated dataset, including the discovery cohorts GSE97508, GSE58780, and GSE111168 and the independent validation cohorts GSE87042 and GSE214685, SHAP values were calculated using the kernelshap and shapviz packages. Feature contributions were visualized using SHAP feature-importance plots, beeswarm plots, dependence plots, and individual sample explanation plots (Qi et al., 2025).

### 2.11 Single-gene gene set enrichment analysis (GSEA) of hub genes

Gene set enrichment analysis was performed for the five hub genes using the clusterProfiler package (v4.4.4). Based on the median expression level of each target gene, RB patient samples were divided into high-expression and low-expression groups. The log2 fold change of all genes between the high- and low-expression groups was then calculated and ranked. GSEA was performed using the KEGG gene set database (c2. cp.kegg.v7.5.1. symbols.gmt), with *P* < 0.05 set as the significance threshold. The top five most significant enriched pathways for each gene were selected and visualized using the enrichplot package (v1.16.2) (Subramanian et al., 2005).

### 2.12 Single-gene gene set variation analysis (GSVA) enrichment analysis of hub genes

GSVA was used to assess pathway activity changes related to the expression levels of the five hub genes. Based on the ssGSEA algorithm and KEGG gene sets, RB samples were divided into high- and low-expression groups according to the median expression of each gene. Differences in pathway activity between the two groups were analyzed using t-tests, and t values were used to measure the degree of change. The results were then shown using bar plots (Xu et al., 2026).

### 2.13 Molecular docking analysis

The molecular structure of TCDD was obtained from PubChem (https://pubchem.ncbi.nlm.nih.gov/), and the target protein structures were obtained from the RCSB PDB database (http://www.rcsb.org/) or the AlphaFold Protein Structure Database (https://alphafold.ebi.ac.uk/), depending on availability. Target-specific docking search spaces were defined using the GetBox Plugin because experimentally validated TCDD-binding sites were unavailable. Molecular docking was performed using AutoDock Vina (v1.1.2), with the exhaustiveness value fixed at 32 and up to 10 binding modes (Rosignoli and Paiardini, 2022). The target-specific grid-box centres and dimensions are provided in Supplementary Table S3. The rationale for protein structure selection and detailed docking procedures are provided in Supplementary Medhods S1 and Supplementary Table S4. The docking protocol was validated by redocking the co-crystallized ligands of CDK1, CA14, and KDR into their native binding pockets and calculating the heavy-atom root-mean-square deviation (RMSD) values between the redocked and crystallographic poses. An RMSD below 2.0 Å was considered to indicate acceptable reproduction of the crystallographic binding pose (Trott and Olson, 2010). The validation results are summarized in Supplementary Table S5. Docking poses were visualized using PyMOL (v2.6.0) (Ravindranath et al., 2015).

### 2.14 Pan-cancer expression patterns, prognostic value, and interaction analysis of the five hub genes

This study performed a systematic pan-cancer analysis of the five hub genes using RNA-seq data from The Cancer Genome Atlas (TCGA, https://portal.gdc.cancer.gov/). Fragments Per Kilobase of transcript per Million mapped reads (FPKM) data for 33 cancer types were obtained from TCGA. Ensembl gene IDs were converted to official gene symbols, and duplicated symbols were merged by mean expression values. Sample types were defined according to sample barcodes, and expression matrices from all cancer types were integrated into a unified pan-cancer dataset (Wu et al., 2025). Boxplots were used to show the expression distribution of the five hub genes across the pan-cancer cohort. For cancer types with at least five normal samples, Wilcoxon rank-sum tests were used to compare gene expression between tumor and normal tissues, and the results were analyzed and visualized using the ggpubr package. Based on the differential expression results, heatmaps were generated using pheatmap to show pan-cancer dysregulation patterns. Pearson correlation analysis was used to assess co-expression relationships among the five hub genes in pan-cancer tumor samples. Correlation coefficient matrices were calculated and visualized using TCGA pan-cancer tumor samples and the corrplot package. Kaplan-Meier analysis was used to assess the relationship between gene expression and survival prognosis, with Log-rank test *P* < 0.05 considered significant, and significant results were visualized using the survminer package (Wu et al., 2025). Univariate Cox regression analysis was used to evaluate the effect of gene expression on survival risk. Hazard ratios (HRs) and 95% confidence intervals were calculated and shown using forest plots.

### 2.15 Association analysis of immune features and the tumor microenvironment

Based on the TCGA pan-cancer cohort, we systematically analyzed the associations of the five hub genes with tumor immune features, microenvironment composition, stemness, and genomic stability. Information on the six immune subtypes (C1-C6) defined by Thorsson et al. (Thorsson et al., 2018), ImmuneScore and StromalScore calculated by the ESTIMATE algorithm, DNA stemness score based on epigenetic features (DNAss), RNA stemness score based on transcriptomic features (RNAss), microsatellite instability (MSI), and tumor mutational burden (TMB) data for each cancer type were obtained from the UCSC Xena database. Gene expression data were uniformly preprocessed and normalized using the limma package. Kruskal–Wallis tests were used to assess differences in gene expression among immune subtypes, and the results were visualized using ggplot2. Spearman correlation analysis was used to examine the associations between gene expression and immune scores, stromal scores, and stemness scores, and cross-cancer correlation heatmaps were generated using corrplot. MSI and TMB were analyzed separately for each of the five genes. Spearman correlations were calculated by cancer type, and radar plots were used to show cancer-specific association patterns for each gene (Kim et al., 2026).

### 2.16 Evaluation of the effects of hub genes on the tumor immune microenvironment

We assessed the associations between the five hub genes and tumor immune microenvironment features in the TCGA pan-cancer cohort. Gene expression data for each cancer type were obtained from the UCSC Xena database, and two computational approaches were integrated: CIBERSORT was used to estimate the relative infiltration proportions of 22 immune cell types, and ESTIMATE was used to calculate ImmuneScore and StromalScore (Wang et al., 2026). During data preprocessing, normal samples were removed, and overlapping samples between gene expression and immune feature data were retained. Stratified Spearman correlation analysis was performed by cancer type. Significant associations were visualized using ggplot2, ggpubr, and ggExtra to generate scatter plots with linear fitting lines and marginal density curves.

### 2.17 Cell culture and transfection

The non-tumor human retinal pigment epithelial cell line ARPE-19 and the RB cell lines Y79 and WERI-RB-1 were purchased from the National Collection of Authenticated Cell Cultures (Shanghai, China). All cell lines were authenticated by STR profiling (Jiangsu Genetic Testing Biotechnology Co., Ltd.) and were routinely tested for *mycoplasma* to ensure contamination-free culture. Cells were maintained at 37 °C in a humidified incubator with 5% CO_2_. ARPE-19 cells were cultured in DMEM/F12 medium (Gibco, United States of America) supplemented with 10% fetal bovine serum (Gibco, China) and 1% penicillin-streptomycin solution (Thermo Fisher, United States of America). Y79 and WERI-RB-1 cells were cultured in RPMI-1640 medium (Solarbio, Beijing, China) supplemented with 20% fetal bovine serum and 1% penicillin-streptomycin solution. The CDK1 overexpression (OE) plasmid and short hairpin RNA (shRNA) plasmid were synthesized by Nanjing Keygen Biotech Co., Ltd. The shRNA target sequences are shown in Supplementary Table S1. RB cells were transfected using Lipofectamine 3,000 (Invitrogen, United States of America) according to the manufacturer’s instructions. After transfection, cells were maintained at 37 °C with 5% CO_2_ and used for subsequent experiments.

### 2.18 RNA extraction and quantitative real-time polymerase chain Reaction (qRT-PCR)

Total RNA was extracted from samples using TRIzol reagent (Invitrogen, United States of America), and RNA concentration and purity were measured with a NanoDrop spectrophotometer. The A260/A280 ratios of all RNA samples ranged from 1.8 to 2.1, indicating that RNA purity was suitable for subsequent experiments. Then, 1 μg of total RNA was reverse-transcribed into cDNA using the PrimeScript™ RT reagent kit (Takara Bio, Japan) according to the manufacturer’s instructions. Quantitative real-time PCR was performed using a SYBR Green qPCR kit on a real-time PCR system (Hongshi, Shanghai, China). The reaction system was prepared according to the kit protocol, and GAPDH was used as the internal reference gene. Each group included at least three biological replicates, and each biological replicate included three technical replicates. The mean Ct value of the technical replicates was used for subsequent analysis. Relative expression levels of target genes were calculated using the 2^-ΔΔCt^ method, and the primer sequences are listed in Table 1.

**TABLE 1:** Primer sequences.

Primer name	Forward primer (5′–3′)	Reverse primer (5′–3′)
CDK1	GGA​TGT​GCT​TAT​GCA​GGA​TTC​C	CAT​GTA​CTG​ACC​AGG​AGG​GAT​AG
CA14	CTA​CAA​TGG​CTC​GCT​CAC​AA	GCT​GAT​TGA​GAG​GCT​GAA​GG
GRIA2	CAC​CCC​ACA​TCG​ACA​ATT​TGG	GAC​GTG​GAG​TGT​TCC​GCA​A
PIP5K1B	TGA​CCC​CAG​CAC​ATC​ACT​AC	GCT​CCA​GGG​TTA​GAC​AGT​TCT
KDR	GCC​AAC​TCT​ATG​GCA​GAA​GC	CTG​AAC​ACC​ATG​CCA​CTG​TC
GAPDH	GGA​GCG​AGA​TCC​CTC​CAA​AAT	GGC​TGT​TGT​CAT​ACT​TCT​CAT​GG

### 2.19 Western blot analysis

Western blot was used to detect target protein expression. Cells were lysed with Radioimmunoprecipitation Assay buffer (RIPA) buffer containing protease inhibitors, and total protein was extracted and quantified using the Bicinchoninic Acid Assay (BCA) method. For each group, 30 µg of total protein was loaded and separated by Sodium Dodecyl Sulfate-Polyacrylamide Gel Electrophoresis (SDS-PAGE) using gels of appropriate concentrations according to the molecular weights of the target proteins. The proteins were then transferred onto Polyvinylidene fluoride (PVDF) membranes. After transfer, the membranes were blocked with rapid blocking buffer (Servicebio, Wuhan, China) and incubated overnight at 4 °C with primary antibodies against CDK1 (Cat. No. 33–1800, 1:1,000, Thermo Fisher) and β-actin (Cat. No. A1978, 1:10,000, Sigma-Aldrich). The next day, the membranes were incubated with the corresponding HRP-conjugated secondary antibodies. Protein signals were detected using an Enhanced Chemiluminescence (ECL) chemiluminescence system, and band intensities were quantified using ImageJ software (v1.53). β-actin was used as the internal control for normalization.

### 2.20 Statistical analysis

Data analysis was performed using GraphPad Prism 9.0 (GraphPad Software, La Jolla, CA, United States of America). All experiments were independently repeated at least three times, and quantitative data are presented as mean ± SEM. Normality and homogeneity of variance were tested before statistical analysis. For data with normal distribution and equal variance, comparisons between two groups were performed using an unpaired two-tailed Student’s t-test. Comparisons among multiple groups were performed using one-way ANOVA. *P* < 0.05 was considered statistically significant.

## 3 Results

### 3.1 Identification of TCDD target proteins

**FIGURE 2:**
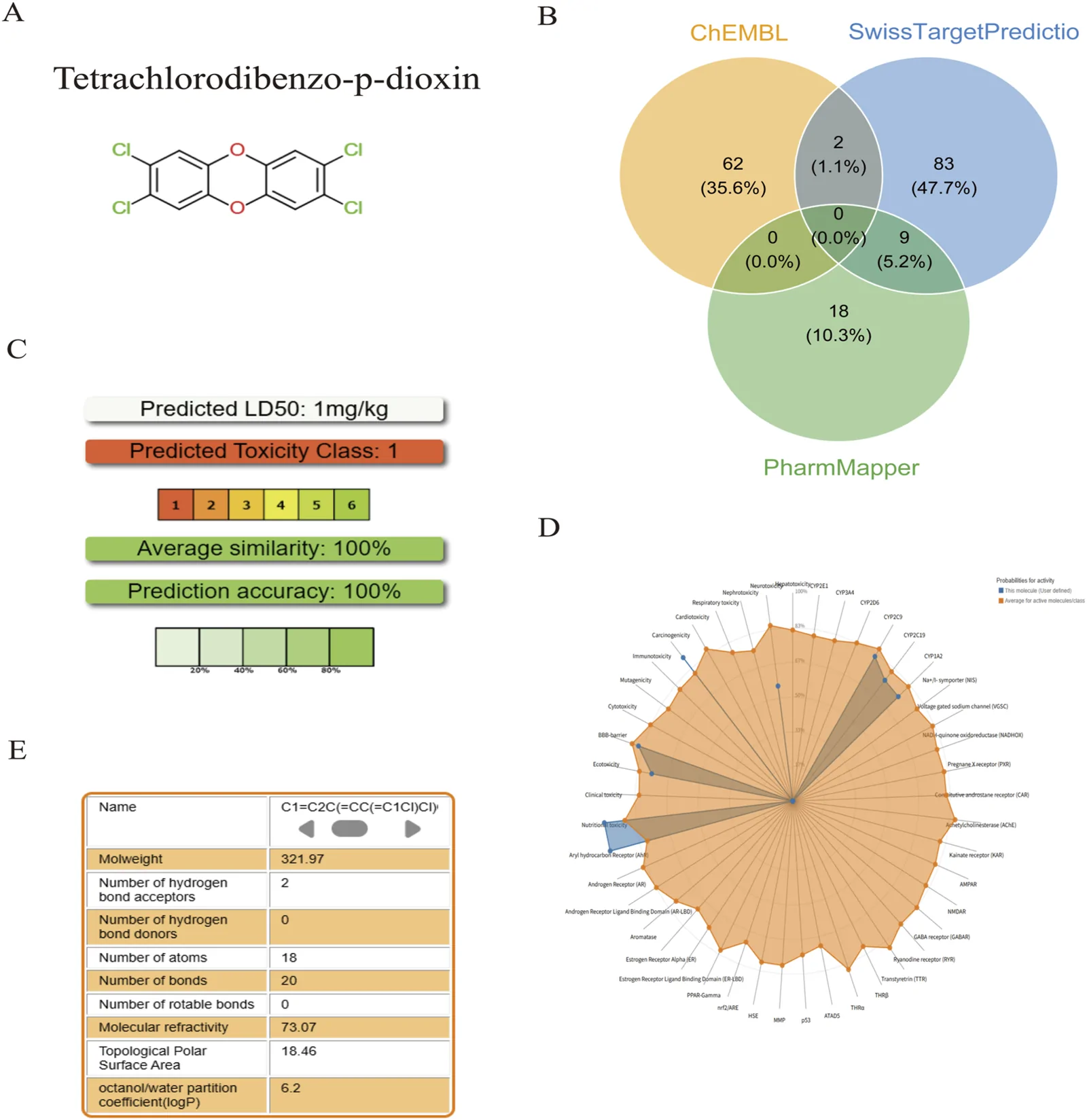
Molecular structure, target prediction, and toxicity analysis of TCDD. **(A)** Molecular structure of TCDD. **(B)** Target prediction using ChEMBL, SwissTargetPrediction, and PharmMapper. **(C)** Toxicity prediction of TCDD. **(D)** Radar plot of TCDD toxicity prediction. **(E)** Physicochemical property assessment of TCDD based on ProTox 3.0.

The molecular structure of TCDD was obtained from PubChem (Figure 2A), and its two-dimensional structure was represented by the SMILES notation: C1 = C2C(=CC(=C1Cl)Cl)OC3 = CC(=C(C=C3O2)Cl)Cl. Potential human targets of TCDD were predicted using ChEMBL, SwissTargetPrediction, and PharmMapper. The resulting target lists were standardized using UniProt and converted into official gene symbols. After data integration and duplicate removal, 174 unique human protein targets were retained (Figure 2B).

### 3.2 Predicted toxicological and physicochemical profiles of TCDD

ADMETlab 3.0 predicted multiple toxicological endpoints for TCDD, including carcinogenicity, genotoxicity, eye irritation, environmental toxicity, hepatotoxicity, neurotoxicity, nephrotoxicity, and cardiotoxicity (Supplementary Material one and Figure 2D). The predicted probability of eye irritation was 0.806. ProTox 3.0 classified TCDD as toxicity class 1 (Figure 2C). Physicochemical property analysis showed that TCDD had a logP value of 6.2, no rotatable bonds, and a topological polar surface area of 18.46 Å^2^, indicating high lipophilicity, structural rigidity, and low polarity (Figure 2E).

### 3.3 Differential expression profiling in RB

**FIGURE 3:**
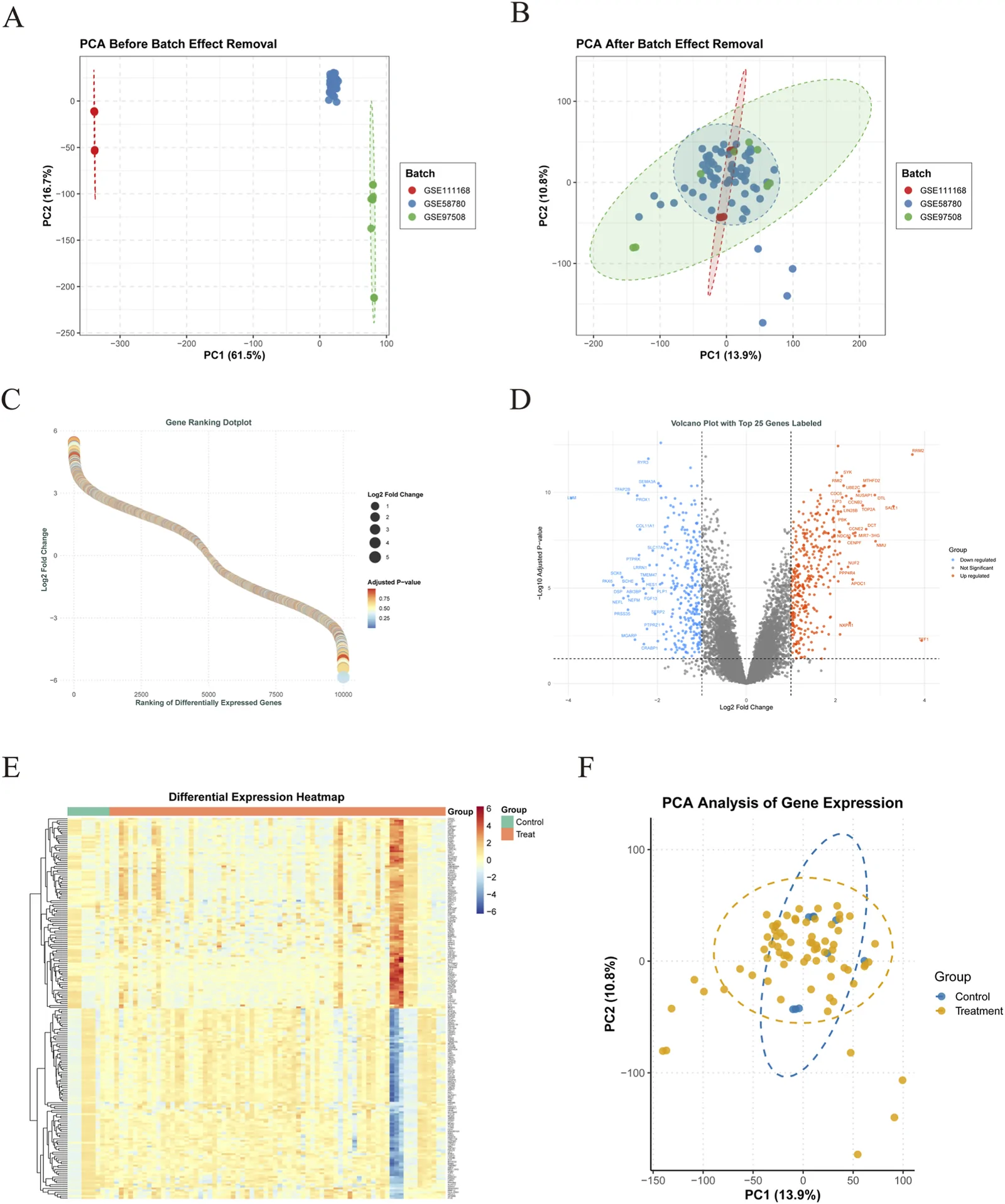
Data integration and differential expression analysis in RB. **(A)** PCA plot before correction, showing sample separation by source dataset. **(B)** PCA plot after correction, showing effective removal of batch effects and uniform mixing of samples. **(C)** Gene ranking dot plot of differentially expressed genes. **(D)** Volcano plot of differentially expressed genes. **(E)** Clustered heatmap of differentially expressed genes. **(F)** PCA analysis based on differentially expressed genes.

This study integrated three RB transcriptomic datasets, GSE97508, GSE58780, and GSE111168, and applied SVA and ComBat algorithms for batch-effect correction and normalization of the merged gene expression matrix. To assess the correction effect, principal component analysis was performed. Before correction, samples from different datasets were clearly separated along PC1 and PC2 (Figure 3A), indicating marked technical variation. After correction, samples from different sources showed a more consistent distribution (Figure 3B), suggesting that batch effects were effectively removed. Based on the criteria of |log_2_FC| > 1 and FDR <0.05, a total of 4,711 genes were identified as significantly differentially expressed in RB. These genes were shown using several visualization methods. The gene ranking dot plot showed the distribution of genes ranked by the degree of differential expression, with a clear association between log_2_FC and adjusted *P* values (Figure 3C). The volcano plot showed the overall distribution of differentially expressed genes (Figure 3D). The heatmap showed the expression patterns of significant DEGs across samples (Figure 3E). To further assess data normalization and group differences, PCA was performed based on the DEGs (Figure 3F). The results showed clear separation between the control and RB groups along PC1 and PC2, further supporting the effectiveness of the data processing workflow and the RB-specific transcriptomic features.

### 3.4 Integrated WGCNA and DEG analysis for identification of RB-Associated genes

**FIGURE 4:**
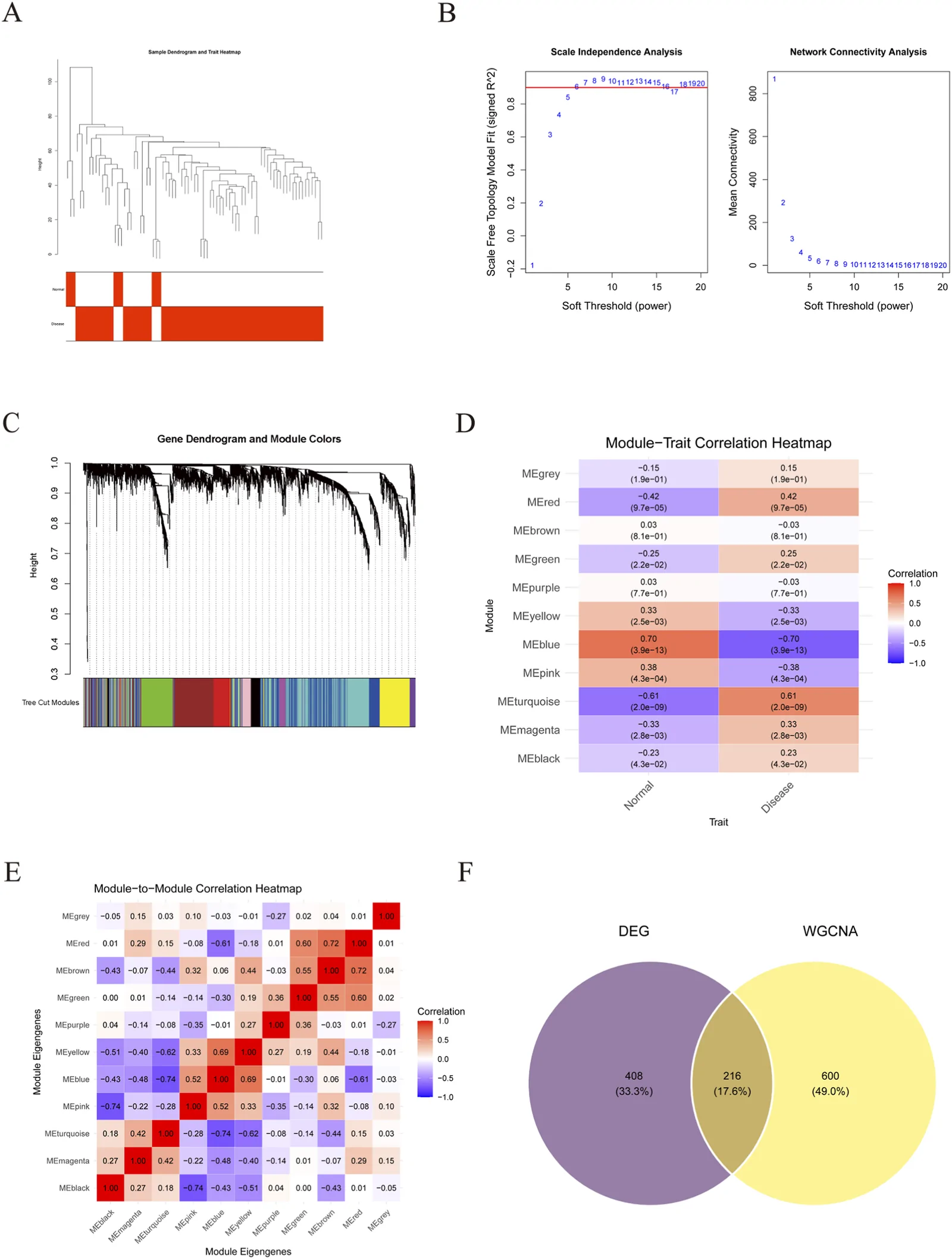
Integrated WGCNA and differential expression analysis for identification of RB-associated genes. **(A)** Hierarchical clustering of control and RB samples. **(B)** Soft-threshold power selection and network connectivity analysis; *β* = 6 was selected. **(C)** Gene dendrogram and module assignment based on dynamic tree cutting. **(D)** Module–trait correlation heatmap; values indicate correlation coefficients and *P* values. **(E)** Heatmap of correlations among co-expression modules. **(F)** Venn diagram showing the overlap between differentially expressed genes and MEblue module genes.

Weighted gene co-expression network analysis was performed to identify RB-associated gene modules. Hierarchical clustering showed differences in sample distribution between the control and RB groups (Figure 4A). Soft-thresholding powers (*β*) from 1 to 20 were evaluated, and *β* = 6 was selected because the scale-free topology fit index reached 0.8 (Figure 4B). Based on this parameter, a topological overlap matrix was constructed, and 11 co-expression modules were identified using the dynamic tree-cut method (Figure 4C). Module–trait correlation analysis showed that the MEblue module had the strongest correlation with RB status (*r* = −0.70, *P* = 3.9 × 10^−13^), and genes in this module were retained for subsequent analysis (Figure 4D). Correlations among the co-expression modules are shown in Figure 4E. Intersection of the MEblue module genes with the differentially expressed genes identified 1,224 overlapping RB-associated genes (Figure 4F).

### 3.5 Functional enrichment and PPI network analysis of overlapping TCDD–RB targets

**FIGURE 5:**
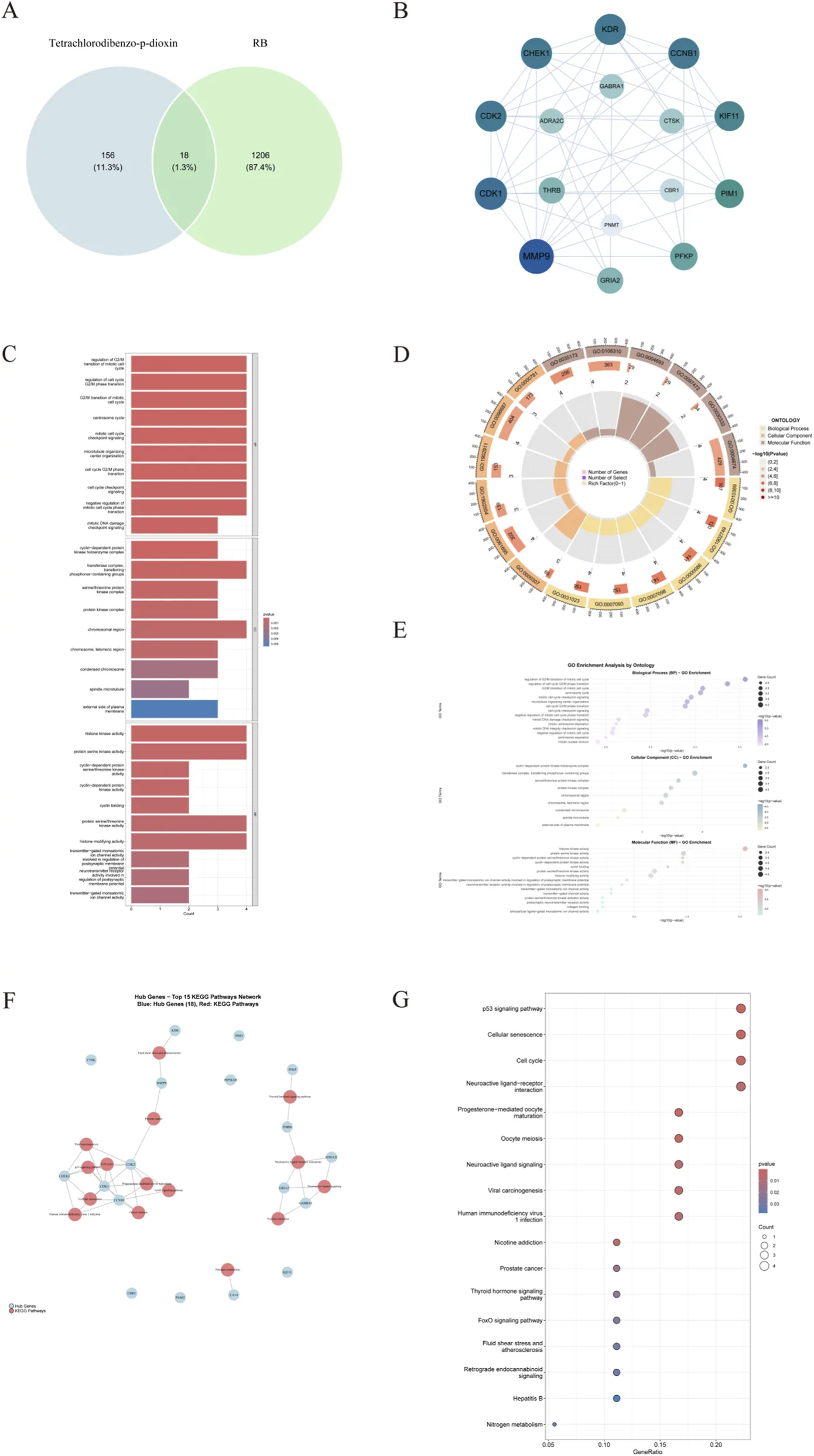
Functional enrichment and PPI network analysis of overlapping TCDD–RB targets. **(A)** Venn diagram showing 18 overlapping targets between predicted TCDD-related targets and RB-related genes. **(B)** Protein–protein interaction network of the 18 overlapping targets; edges represent predicted functional associations. **(C)** GO enrichment analysis of biological process, cellular component, and molecular function terms. The x-axis indicates gene count, and the color gradient represents adjusted *P* values. **(D)** Circular plot of representative GO terms. The outer ring shows GO terms and identifiers, the middle bars indicate enriched gene counts, and the inner color gradient represents enrichment significance. **(E)** Bubble plot of GO enrichment results; bubble size indicates gene count, and color intensity represents enrichment significance. **(F)** Target–KEGG pathway network. Blue nodes represent overlapping targets, red nodes represent significantly enriched pathways, and edges indicate target–pathway associations. **(G)** Bubble plot of KEGG enrichment results; bubble size indicates gene count, color intensity represents enrichment significance, and the x-axis shows the gene ratio.

By intersecting the predicted TCDD targets with RB-related genes, 18 overlapping targets were identified (Figure 5A). A protein–protein interaction network was subsequently constructed to examine the interactions among these targets. The network showed extensive interactions among the target proteins, and topology analysis identified several highly connected nodes, including CDK1 (Figure 5B).

GO enrichment analysis showed that the overlapping targets were significantly enriched in biological process terms, including “regulation of mitotic cell cycle G2/M phase transition,” “cell cycle G2/M phase transition,” and “centrosome cycle.” Enriched cellular component terms included “cyclin-dependent protein kinase holoenzyme complex” and “serine/threonine protein kinase complex,” whereas enriched molecular function terms included “histone kinase activity,” “cyclin binding,” and “protein serine/threonine kinase activity” (Figures 5C–E).

KEGG enrichment analysis showed significant enrichment in several pathways, including the cell cycle, p53 signaling pathway, cellular senescence, thyroid hormone signaling pathway, and neuroactive ligand–receptor interaction pathway (Figures 5F,G).

### 3.6 Identification of hub genes associated with TCDD and RB

**FIGURE 6:**
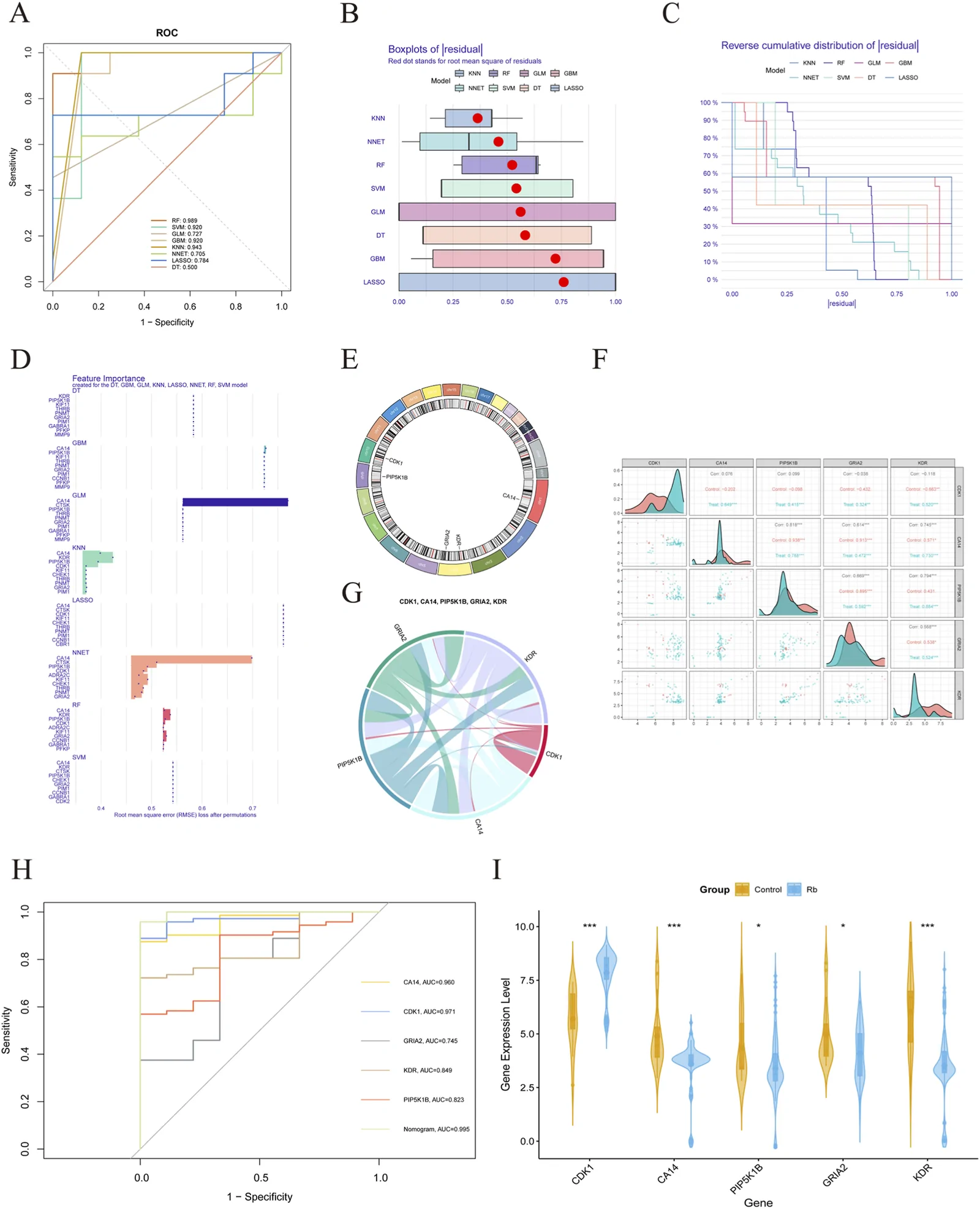
Identification and characterization of hub genes associated with TCDD and RB. **(A)** ROC curves of the eight machine-learning models. RF showed the highest AUC. **(B)** Distribution of absolute residuals across the models. **(C)** Reverse cumulative distribution of absolute residuals. **(D)** Multi-model feature-importance ranking and permutation-based RMSE loss. **(E)** Chromosomal locations of the five hub genes. **(F)** Correlations between CDK1 and the other hub genes in the control and RB groups. **(G)** Interaction network among CDK1, CA14, PIP5K1B, GRIA2, and KDR. **(H)** ROC analysis of the individual hub genes and the integrated five-gene model. **(I)** Expression distributions of the five hub genes in the control and RB groups. Yellow represents the control group, and blue represents the RB group. *P* < 0.05, P < 0.01, and *P* < 0.001.

Through systematic machine learning analysis of the 18 candidate genes, we constructed eight prediction models to identify hub genes associated with TCDD and RB. RF achieved the highest ROC AUC and showed strong discriminatory performance with relatively stable residuals. It was therefore selected as the final model (Figures 6A–C). Based on the feature-importance ranking of the selected RF model, five hub genes were identified: CA14, PIP5K1B, GRIA2, KDR, and CDK1. Multi-model feature-importance analysis further supported the predictive relevance of these genes (Figure 6D). To assess their discriminatory performance, ROC curve analysis was performed (Figure 6H). CDK1 (AUC = 0.971) and CA14 (AUC = 0.960) showed excellent discriminatory ability, while KDR (AUC = 0.849) and PIP5K1B (AUC = 0.823) also showed discriminatory performance. The model integrating all five hub genes achieved the highest AUC of 0.995. Chromosomal localization analysis showed the genomic positions of the five hub genes (Figure 6E). Correlation analysis showed that, in the RB group, CDK1 was significantly positively correlated with CA14, PIP5K1B, GRIA2, and KDR, with correlation coefficients of 0.649, 0.415, 0.324, and 0.520, respectively (*P* < 0.01), while negative correlation trends were observed in the control group (Figure 6F). The hub gene interaction network (Figure 6G) further showed that CDK1 was located at the center of the network and connected to the other four genes. The violin plots in Figure 6I show the expression levels of the five hub genes in the normal control and RB groups. Compared with the control group, CDK1 was significantly upregulated in RB tissues, whereas CA14, PIP5K1B, GRIA2, and KDR showed downregulated expression.

**FIGURE 7:**
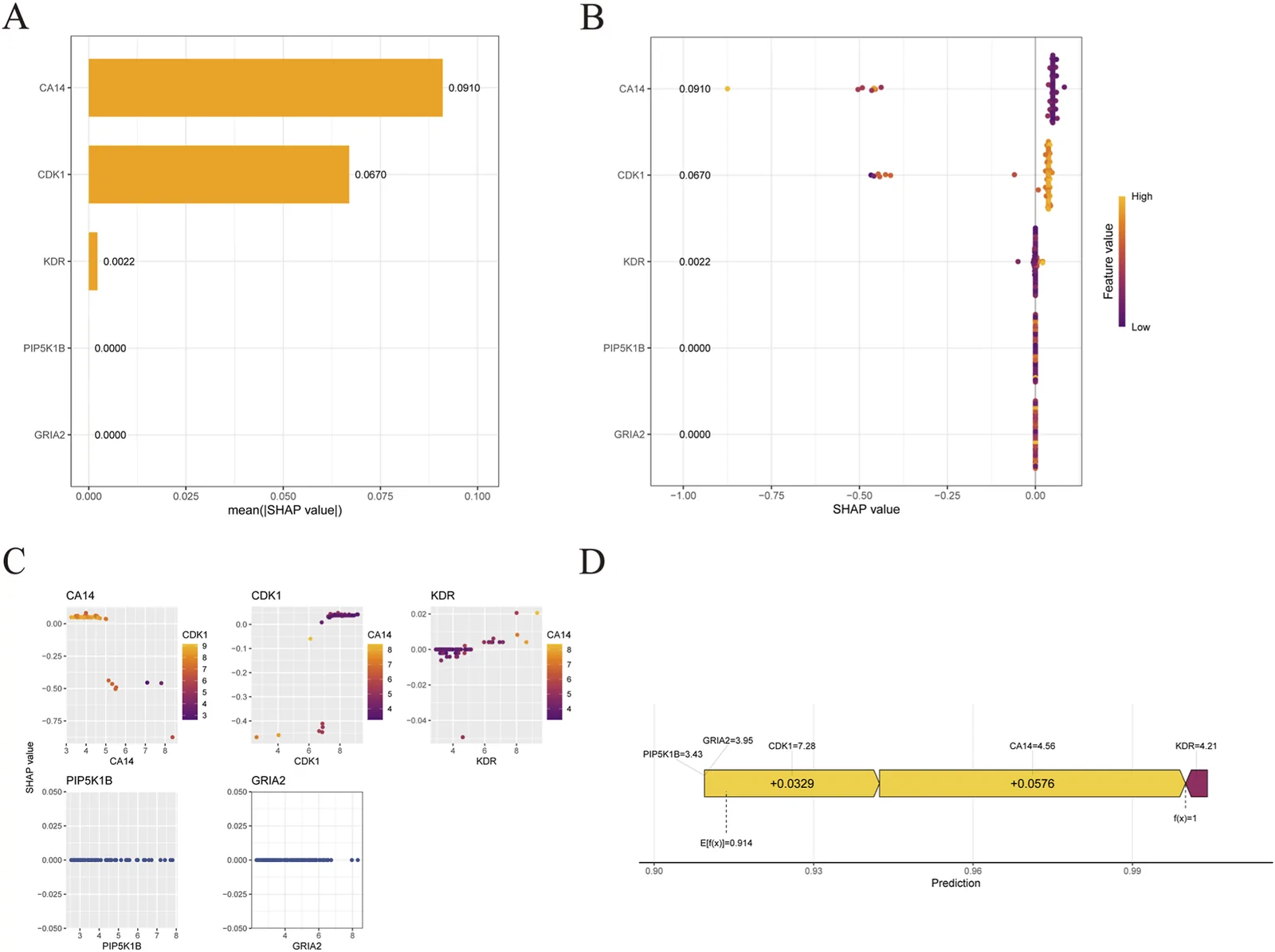
SHAP interpretability analysis of the RF model. **(A)** SHAP feature-importance plot based on mean absolute SHAP values. CA14 and CDK1 showed the highest contributions. **(B)** SHAP beeswarm plot showing the distribution of feature contributions across samples. Point color represents the corresponding feature value. **(C)** SHAP dependence plots showing the relationships between gene expression and SHAP values. **(D)** SHAP explanation plot for an individual prediction showing the contribution of each gene to the model output.

SHAP analysis was performed to assess the contribution of each hub gene to the RF model. CA14 and CDK1 showed the highest mean absolute SHAP values, at 0.091 and 0.067, respectively, whereas KDR showed a lower contribution of 0.0022, and PIP5K1B and GRIA2 showed minimal contributions in the final model (Figure 7A). The SHAP beeswarm plot showed the distributions of feature values and their prediction contributions (Figure 7B). The SHAP dependence plots showed feature-dependent relationships between gene expression and SHAP values (Figure 7C). In the representative individual prediction, CA14 and CDK1, with feature values of 4.56 and 7.28, respectively, made the largest positive contributions, resulting in a predicted value of *f*(x) = 1 compared with the baseline expected value of *E* [*f*(x)] = 0.914 (Figure 7D).

### 3.7 Single gene GSEA and GSVA enrichment analysis

**FIGURE 8:**
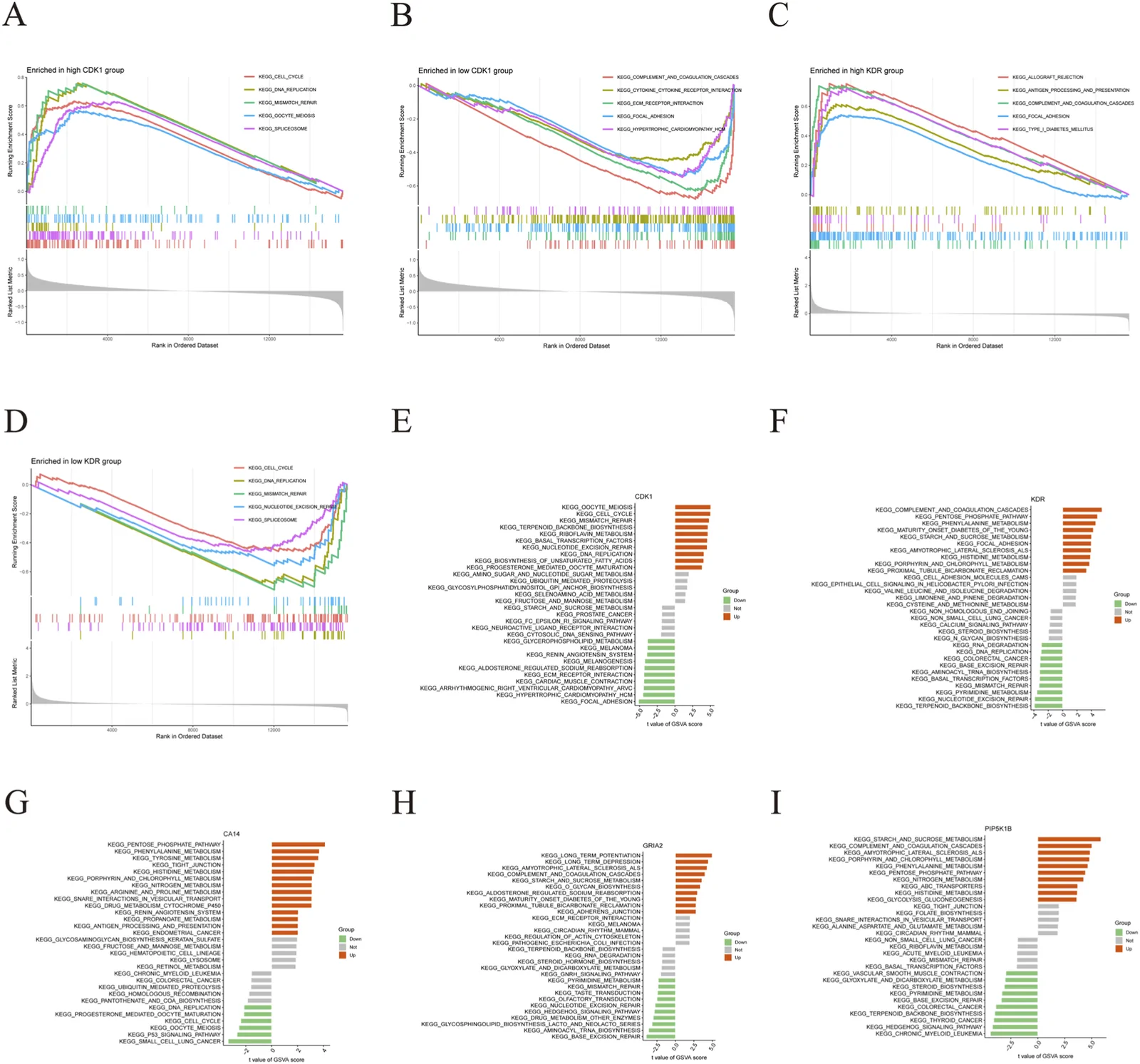
**(A–D)** Single-gene GSEA enrichment analysis. **(E–I)** Single-gene GSVA enrichment analysis. The five hub genes are mainly involved in biological processes related to cell cycle regulation, DNA replication and damage repair, metabolic reprogramming, neural signaling, and tumor microenvironment regulation.

Single-gene GSEA showed that the five hub genes were associated with distinct pathways in RB. High CDK1 expression was significantly enriched in cell cycle and DNA replication pathways, whereas low CDK1 expression was enriched in complement and coagulation cascades (Figures 8A,B). The CA14 high-expression group was mainly enriched in the pentose phosphate pathway and phenylalanine metabolism, whereas the low-expression group was enriched in cell cycle-related pathways (Supplementary Figure S1A-B). High KDR expression was enriched in allograft rejection and antigen presentation pathways, whereas low KDR expression was enriched in cell cycle-related pathways (Figures 8C,D). High GRIA2 expression was associated with the chemokine signaling pathway, whereas low GRIA2 expression was associated with base excision repair (Supplementary Figure S1C-D). High PIP5K1B expression was enriched in complement and coagulation cascades, whereas low PIP5K1B expression was enriched in the cell cycle pathway (Supplementary Figure S1E-F). Overall, the five hub genes showed distinct associations with proliferation-related, metabolic, DNA-repair, and immune-related pathways.

GSVA revealed gene-specific pathway score patterns for the five hub genes in RB. Samples with high CDK1 expression showed higher GSVA scores for cell cycle, DNA replication, and mismatch repair pathways (Figure 8E). High KDR expression was associated with higher scores for complement and coagulation cascades, whereas low KDR expression was associated with DNA replication and repair pathways (Figure 8F). GRIA2 expression was associated with long-term potentiation, amyotrophic lateral sclerosis, and complement-related pathways (Figure 8H). CA14 (Figure 8G) and PIP5K1B (Figure 8I) showed similar GSVA patterns. High expression of both genes was associated with higher scores for the pentose phosphate pathway and phenylalanine metabolism, whereas low expression was associated with higher scores for the cell cycle pathway. These results showed gene-specific associations with proliferation-related, metabolic, DNA-repair, neural-signaling, and complement-related pathways.

### 3.8 Molecular docking analysis of TCDD–hub protein interactions

**FIGURE 9:**
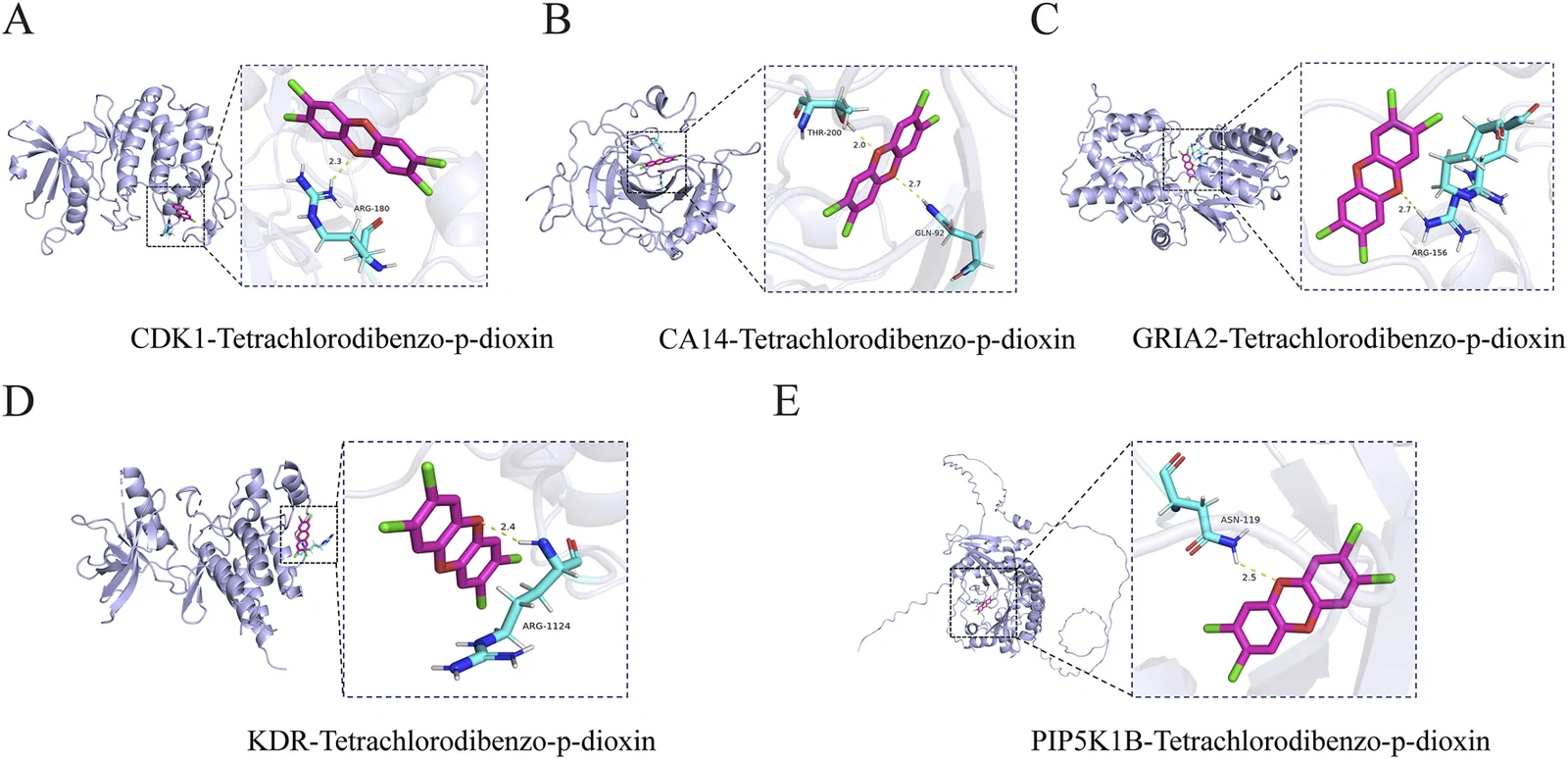
Molecular docking results of TCDD with hub target proteins. **(A)** Docking of TCDD with CDK1 (−8.3 kcal/mol). **(B)** Docking of TCDD with CA14 (−7.4 kcal/mol). **(C)** Docking of TCDD with GRIA2 (−6.8 kcal/mol). **(D)** Docking of TCDD with KDR (−6.9 kcal/mol). **(E)** Docking of TCDD with PIP5K1B (−6.8 kcal/mol). The figure shows the predicted docking positions of TCDD within the protein surface cavities and its predicted spatial relationships with surrounding amino acid residues.

Molecular docking was performed to evaluate the predicted structural compatibility of TCDD with CDK1, CA14, PIP5K1B, GRIA2, and KDR. TCDD showed favorable predicted docking energies for all five proteins, ranging from −8.3 to −6.8 kcal/mol (Figure 9; Table 2). CDK1 showed the most favorable predicted docking energy of −8.3 kcal/mol, followed by CA14 (−7.4 kcal/mol), KDR (−6.9 kcal/mol), GRIA2 (−6.8 kcal/mol), and PIP5K1B (−6.8 kcal/mol). Visualization revealed plausible docking poses of TCDD within the respective protein cavities.

**TABLE 2:** Predicted docking energies of TCDD with the five hub proteins.

Receptor	Ligand	Bind. Energy [kcal/mol]
CDK1	2.3,7,8-Tetrachlorodibenzo-P-Dioxin	−8.3
CA14	2.3,7,8-Tetrachlorodibenzo-P-Dioxin	−7.4
GRIA2	2.3,7,8-Tetrachlorodibenzo-P-Dioxin	−6.8
KDR	2.3,7,8-Tetrachlorodibenzo-P-Dioxin	−6.9
PIP5K1B	2.3,7,8-Tetrachlorodibenzo-P-Dioxin	−6.8

Redocking of the co-crystallized ligands yielded heavy-atom RMSD values of 0.5999 Å for CDK1, 1.8680 Å for CA14, and 0.6989 Å for KDR. All values were below the predefined threshold of 2.0 Å. Superposition of the redocked and crystallographic ligand poses showed close agreement in the corresponding binding pockets (Supplementary Figure S2). Together, these results support the ability of the docking protocol to reproduce the experimentally determined binding poses (Supplementary Table S5). These findings support the use of the docking protocol for computational prioritization but do not establish direct TCDD–protein binding or functional interaction.

### 3.9 Pan-cancer expression, correlation, and prognostic analyses of the five hub genes

**FIGURE 10:**
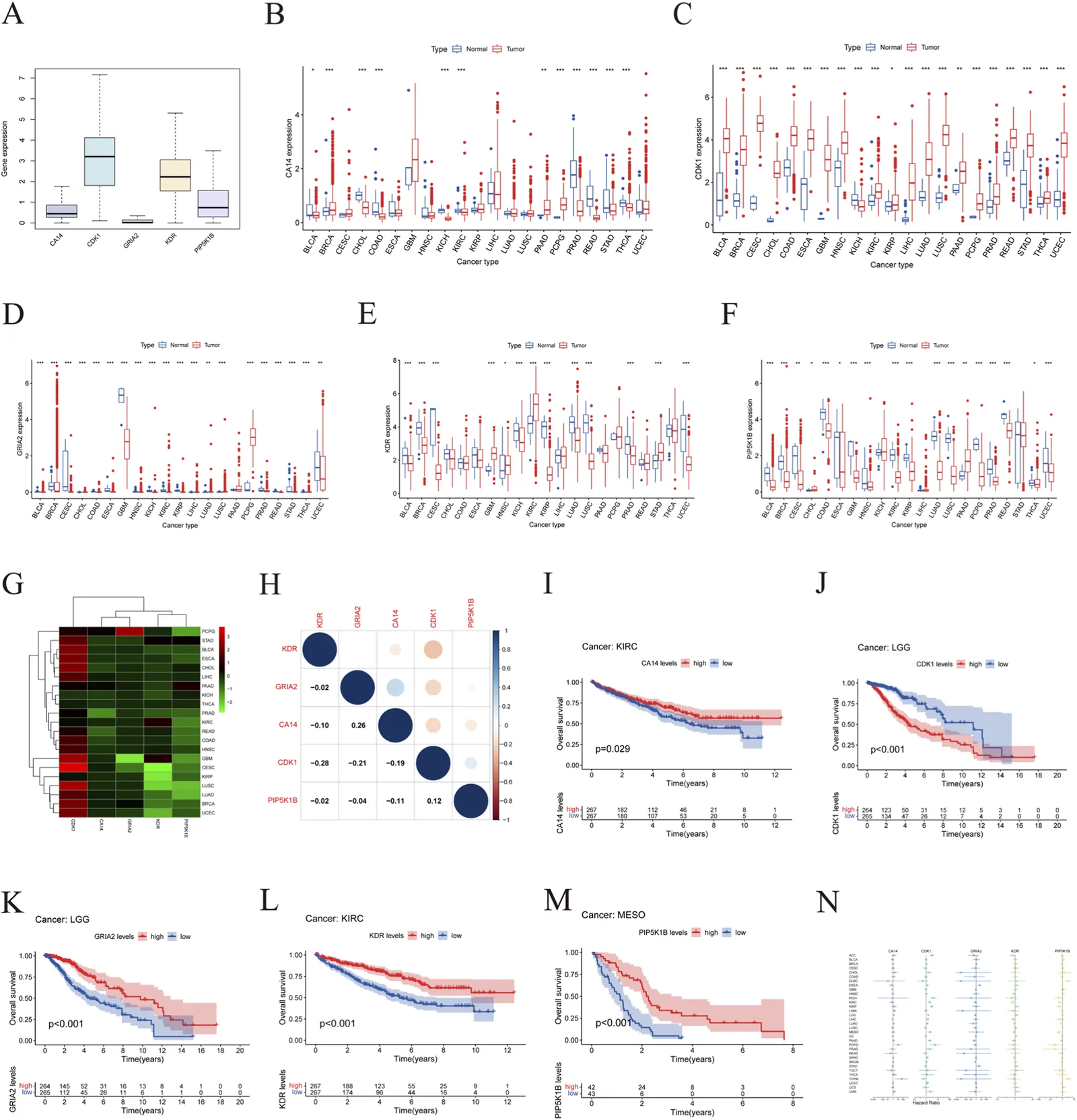
Pan-cancer expression patterns, prognostic value, and interaction analysis of the five hub genes. **(A)** Boxplot showing pan-cancer gene expression levels. The x-axis represents gene names, and the y-axis represents gene expression levels. **(B–F)** Expression comparison of hub genes between tumor and normal samples. Blue represents normal samples, and red represents tumor samples. *P < 0.05, **P < 0.01, ***P < 0.001. **(G)** Heatmap of pan-cancer differentially expressed genes. **(H)** Gene correlation analysis. **(I–M)** Kaplan-Meier analysis of the association between hub gene expression and overall survival (OS). (N) Forest plot showing OS-related associations across 33 cancer types.

Pan-cancer profiling across 33 TCGA cancer types revealed distinct expression patterns of the five RB-associated hub genes. CDK1 showed relatively high expression and was significantly upregulated in multiple tumor types, including breast invasive carcinoma (BRCA), cervical squamous cell carcinoma and endocervical adenocarcinoma (CESC), cholangiocarcinoma (CHOL), colon adenocarcinoma (COAD), and glioblastoma multiforme (GBM). In contrast, CA14, GRIA2, KDR, and PIP5K1B were predominantly downregulated across multiple tumor types, although their expression patterns varied among cancer types (Figures 10A–G). Inter-gene correlation analysis showed generally weak correlations among the five genes across pan-cancer samples (Figure 10H).

Kaplan-Meier analysis showed that high CDK1 expression was associated with poorer overall survival in brain lower-grade glioma (LGG) and adrenocortical carcinoma (ACC). Higher expression of CA14 in kidney renal clear cell carcinoma (KIRC) and GBM, GRIA2 in LGG and ACC, KDR in KIRC and acute myeloid leukaemia (LAML), and PIP5K1B in mesothelioma (MESO) and KIRC was associated with better overall survival (Figures 10I–M; Supplementary Figures S3-S4). Univariate Cox regression analysis further identified cancer-type-specific associations between the expression of the five hub genes and overall survival. Detailed tumor-specific expression and prognostic results are provided in Supplementary Results S1.

### 3.10 Association patterns of hub genes with immune microenvironment features and genomic stability

**FIGURE 11:**
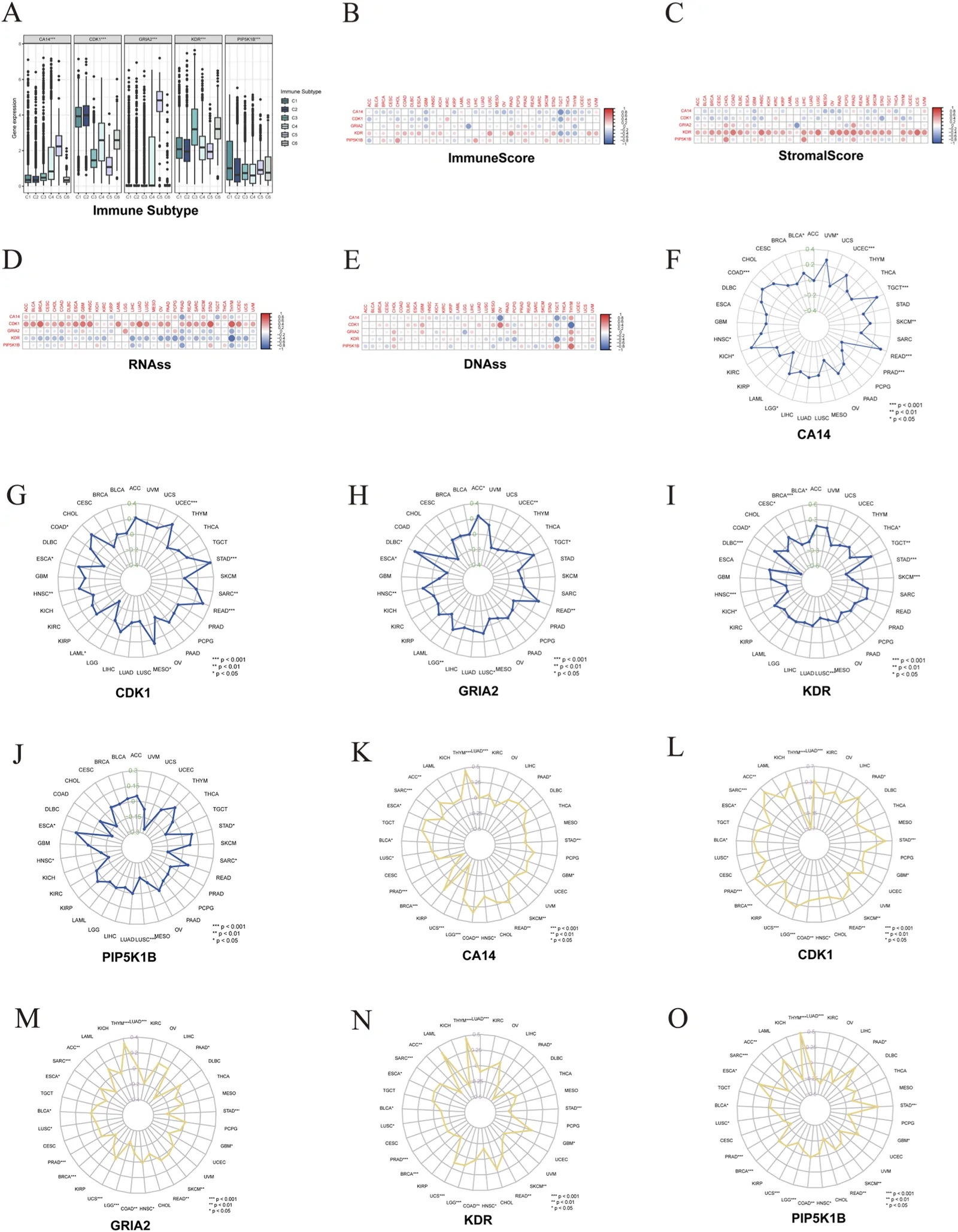
Associations of hub gene expression with immune subtypes, tumor microenvironment scores, stemness scores, MSI, and TMB. **(A)** Expression distributions of the five hub genes across six immune subtypes. **(B–E)** Spearman correlations of hub-gene expression with ImmuneScore, StromalScore, RNA stemness score (RNAss), and DNA stemness score (DNAss) across cancer types. The colour scale represents correlation coefficients. **(F–J)** Spearman correlations between hub-gene expression and microsatellite instability (MSI). **(K–O)** Spearman correlations between hub-gene expression and tumor mutational burden (TMB). In the radar plots, each axis represents a cancer type and axis length indicates the correlation coefficient.

The expression levels of all five hub genes differed significantly across the six immune subtypes (*P* < 0.001) and showed subtype-specific distributions (Figure 11A). CDK1 and PIP5K1B showed relatively high expression in the C1 and C2 subtypes, KDR showed relatively high expression in the C3 subtype, and CA14 and GRIA2 showed relatively high expression in the C5 subtype. Pan-cancer correlation analysis was subsequently performed to examine the associations of hub-gene expression with ImmuneScore, StromalScore, RNA stemness score (RNAss), and DNA stemness score (DNAss) (Figures 11B–E). CA14 showed predominantly negative correlations with ImmuneScore and StromalScore, whereas its correlations with RNAss and DNAss were generally weak. CDK1 showed negative correlations with ImmuneScore and StromalScore and positive correlations with RNAss and DNAss across multiple cancer types. GRIA2 exhibited cancer-type-specific correlations with ImmuneScore and StromalScore, without consistent correlations with either stemness score. KDR showed predominantly positive correlations with ImmuneScore, generally weaker correlations with StromalScore, and weak negative correlations with RNAss and DNAss. PIP5K1B showed predominantly positive correlations with StromalScore, whereas its correlations with ImmuneScore varied across cancer types and no consistent correlations were observed with RNAss or DNAss.

The correlations between hub-gene expression and microsatellite instability (MSI) were cancer-type specific (Figures 11F–J). CA14 was positively correlated with MSI in COAD, rectum adenocarcinoma (READ), and uterine corpus endometrial carcinoma (UCEC). CDK1 was positively correlated with MSI in stomach adenocarcinoma (STAD), UCEC, and READ (*P* < 0.001). KDR was negatively correlated with MSI in head and neck squamous cell carcinoma (HNSC), STAD, and lymphoid neoplasm diffuse large B-cell lymphoma (DLBC) (*P* < 0.001), but positively correlated with MSI in testicular germ cell tumors (TGCT) (*P* < 0.01). GRIA2 was negatively correlated with MSI in UCEC and oesophageal carcinoma (ESCA) and positively correlated with MSI in ACC and DLBC. PIP5K1B was negatively correlated with MSI in lung squamous cell carcinoma (LUSC) (*P* < 0.001) and positively correlated with MSI in ESCA and STAD.

Pan-cancer analysis also identified cancer-type-specific correlations between hub-gene expression and tumor mutational burden (TMB) (Figures 11K–O). CDK1 was positively correlated with TMB in more than 20 cancer types, including STAD, prostate adenocarcinoma (PRAD), and BRCA (*P* < 0.001). GRIA2 was negatively correlated with TMB in several cancer types, including BRCA, STAD, and LGG. KDR was negatively correlated with TMB in BRCA, STAD, and pancreatic adenocarcinoma (PAAD) (*P* < 0.001). CA14 was positively correlated with TMB in thymoma (THYM), COAD, and READ, but negatively correlated with TMB in PRAD and BRCA. PIP5K1B was positively correlated with TMB in THYM, ESCA, and STAD and negatively correlated with TMB in lung adenocarcinoma (LUAD) and sarcoma (SARC). Overall, the direction and magnitude of the correlations with MSI and TMB varied across genes and cancer types.

### 3.11 Associations of hub genes with immune-cell infiltration and tumor microenvironment scores

**FIGURE 12:**
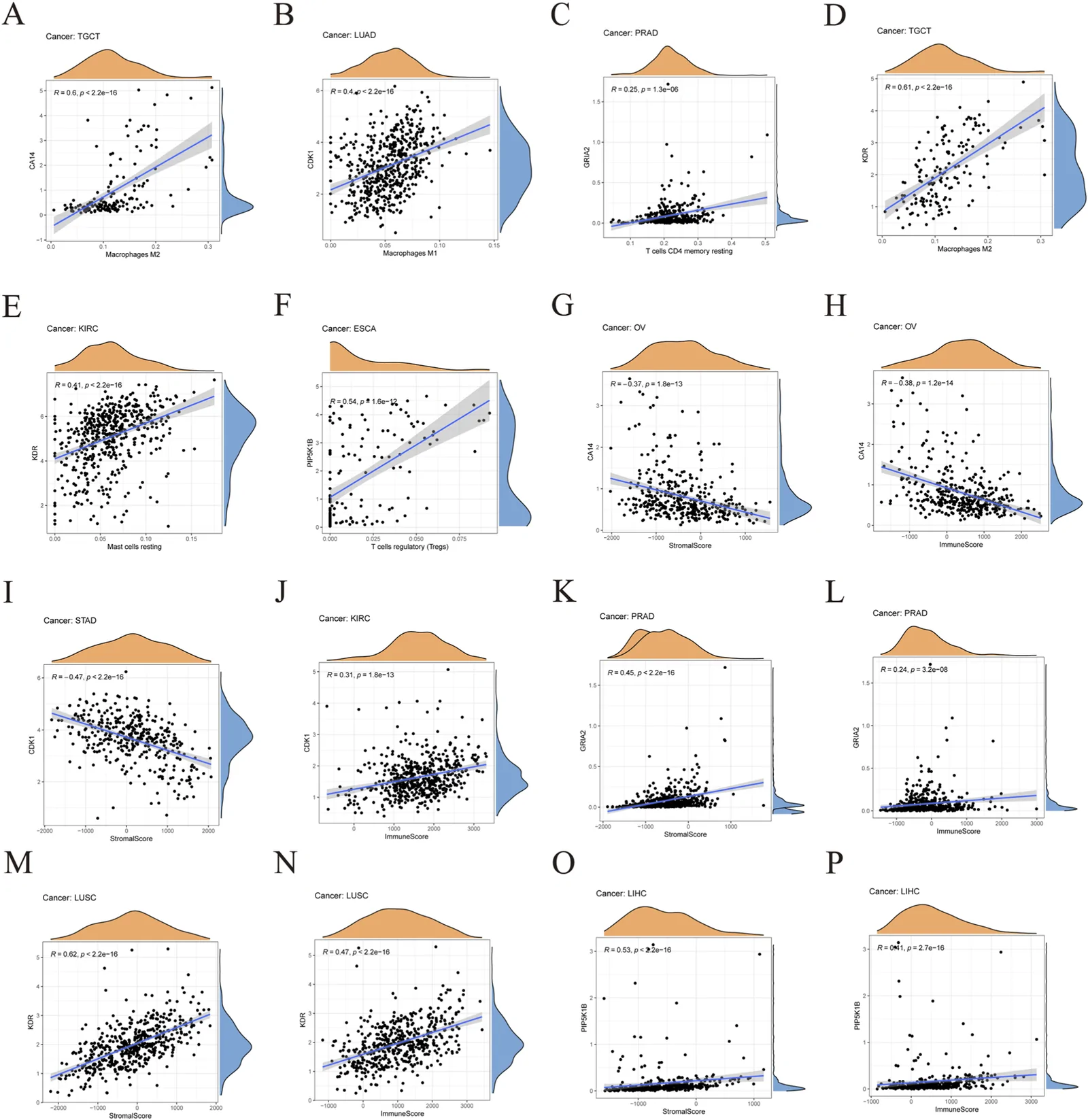
Representative correlations of the five hub genes with immune-cell infiltration and tumor microenvironment scores. **(A)** Correlation between PIP5K1B expression and regulatory T-cell infiltration in ESCA. **(B)** Correlation between CA14 expression and M2 macrophage infiltration in TGCT. **(C)** Correlation between CDK1 expression and M1 macrophage infiltration in LUAD. **(D)** Correlation between GRIA2 expression and resting memory CD4^+^ T-cell infiltration in PRAD. **(E)** Correlation between KDR expression and resting mast-cell infiltration in KIRC. **(F)** Correlation between KDR expression and M2 macrophage infiltration in TGCT. **(G,H)** Correlations of CA14 expression with StromalScore and ImmuneScore in OV. **(I,J)** Correlations of CDK1 expression with StromalScore in STAD and ImmuneScore in KIRC. **(K,L)** Correlations of GRIA2 expression with StromalScore and ImmuneScore in PRAD. **(M,N)** Correlations of KDR expression with StromalScore and ImmuneScore in LUSC. **(O,P)** Correlations of PIP5K1B expression with StromalScore and ImmuneScore in LIHC.

Correlation analysis revealed significant relationships between the expression of the five hub genes and immune-cell infiltration across multiple cancer types (Figures 12A–F; Supplementary Figures S5-S9). CA14 and KDR were positively correlated with M2 macrophage infiltration in TGCT. CDK1 was positively correlated with M1 macrophage infiltration in LUAD, KDR with resting mast-cell infiltration in KIRC, GRIA2 with resting memory CD4^+^ T-cell infiltration in PRAD, and PIP5K1B with regulatory T-cell infiltration in ESCA (*r* = 0.54, *P* < 1.6 × 10^−12^). Negative correlations were observed between KDR or CA14 expression and CD8^+^ T-cell or activated memory CD4^+^ T-cell infiltration in selected cancer types. PIP5K1B and CA14 were negatively correlated with dendritic-cell infiltration, whereas KDR, GRIA2, and CDK1 were negatively correlated with activated natural killer cell infiltration.

The correlations of hub-gene expression with ImmuneScore and StromalScore were further examined across cancer types (Supplementary Figures S10-S14). CA14 was negatively correlated with ImmuneScore (*r* = −0.38) and StromalScore (*r* = −0.37) in OV, with similar negative correlations observed in BLCA and BRCA. CDK1 was negatively correlated with StromalScore in STAD (*r* = −0.47) but positively correlated with ImmuneScore in KIRC (*r* = 0.31). GRIA2 was positively correlated with ImmuneScore (*r* = 0.30) and StromalScore (*r* = 0.45) in PRAD but negatively correlated with both scores in LGG. KDR was positively correlated with ImmuneScore (*r* = 0.47) and StromalScore (*r* = 0.62) in LUSC, with positive correlations also observed in several other cancer types. PIP5K1B was positively correlated with ImmuneScore (*r* = 0.41) and StromalScore (*r* = 0.53) in LIHC but negatively correlated with StromalScore in PAAD. Overall, the direction and magnitude of these correlations varied across hub genes and cancer types.

### 3.12 Experimental validation

**FIGURE 13:**
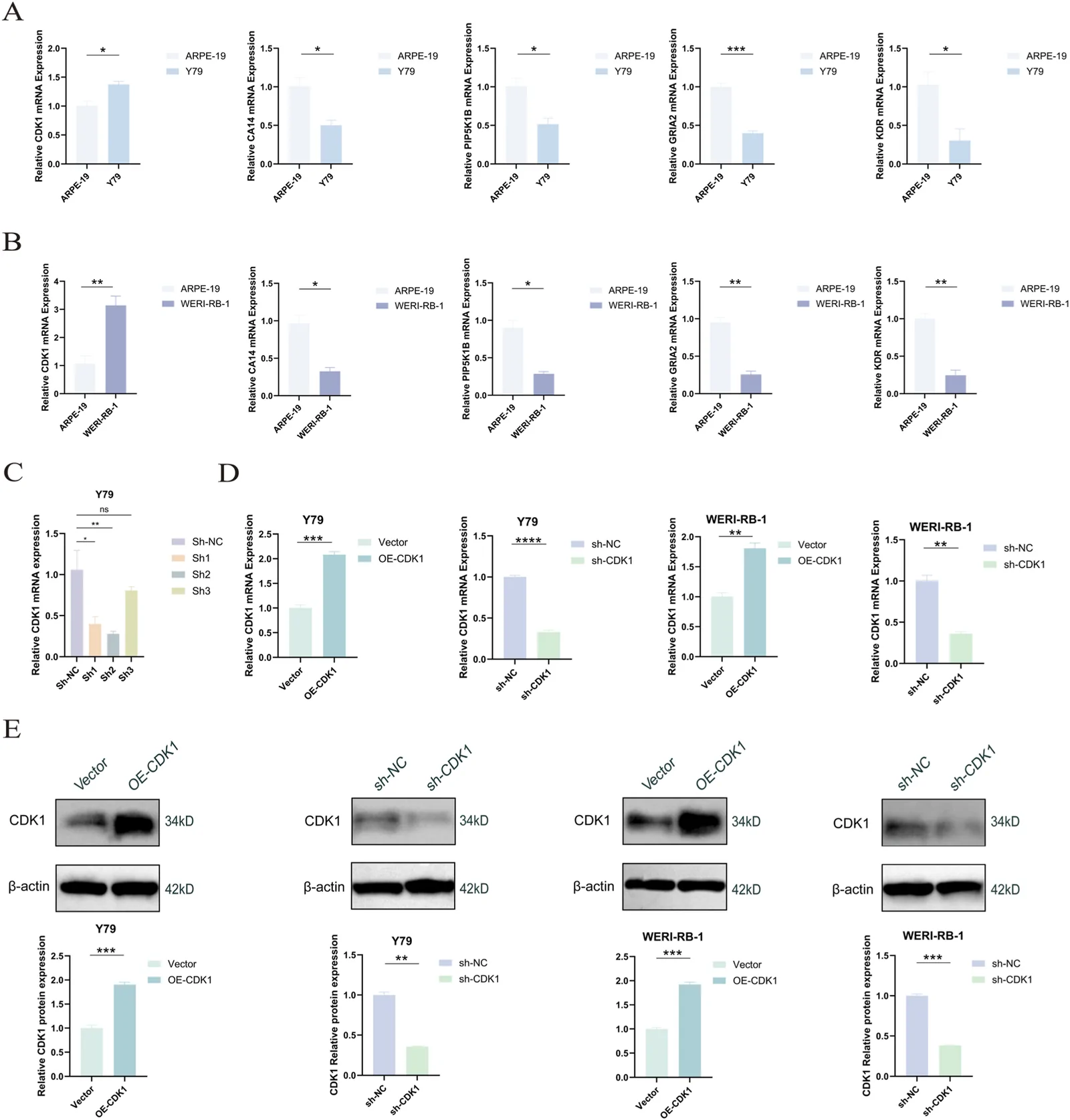
Experimental validation of hub-gene expression and CDK1 overexpression and knockdown in RB cells. **(A,B)** qRT-PCR analysis of the relative mRNA expression levels of the five hub genes in ARPE-19, Y79, and WERI-RB-1 cells. **(C)** qRT-PCR evaluation of CDK1 knockdown efficiency using three shRNA sequences in Y79 cells. **(D)** Relative CDK1 mRNA expression in Y79 and WERI-RB-1 cells following CDK1 overexpression or knockdown. **(E)** Western blot analysis of CDK1 protein expression following CDK1 overexpression or knockdown. Data are presented as the mean ± SEM, *n* = 3. *P* < 0.05, *P* < 0.01, and *P* < 0.001.

qRT-PCR was used to detect the mRNA expression levels of the five hub genes in ARPE-19 cells, which were used as a non-tumor retinal pigment epithelial reference, and the RB cell lines Y79 and WERI-RB-1. Compared with ARPE-19 reference cells, CDK1 mRNA expression was significantly increased in Y79 and WERI-RB-1 cells, while CA14, PIP5K1B, GRIA2, and KDR mRNA expression was significantly decreased. The qRT-PCR expression patterns (Figures 13A,B) were directionally consistent with the GEO-derived expression patterns shown in Figure 6I.

To screen an effective CDK1 knockdown sequence, three shRNAs targeting CDK1 were used in Y79 cells and named sh1, sh2, and sh3. qRT-PCR was then performed to assess the effects of different shRNA sequences on CDK1 mRNA expression. The results showed that sh2 had the highest knockdown efficiency; therefore, sh2 was selected as the CDK1 knockdown sequence for subsequent experiments (Figure 13C).

To confirm the efficiency of CDK1 overexpression and knockdown, we further detected CDK1 mRNA and protein expression in Y79 and WERI-RB-1 cells. qRT-PCR showed that CDK1 mRNA expression was significantly increased in the OE-CDK1 group compared with the Vector group. Compared with the sh-NC group, CDK1 mRNA expression was significantly decreased in the sh-CDK1 group (Figure 13D). Western blotting further showed that CDK1 protein expression was significantly increased in the OE-CDK1 group compared with the Vector group, while it was significantly decreased in the sh-CDK1 group compared with the sh-NC group (Figure 13E). The full, uncropped Western blot membrane images corresponding to Figure 13E are provided in Supplementary Figure S16.

## 4 Discussion

RB is the most common ocular malignancy in infants and young children. Its classical pathogenesis is traditionally thought to be mainly driven by biallelic inactivation of the RB1 gene and abnormal disruption of the downstream RB1-E2F cell cycle axis (Bockus et al., 2025). However, how exogenous environmental factors are associated with this malignant process remains unclear. TCDD is a persistent organic pollutant with systemic toxicity and carcinogenic potential, but whether TCDD-associated molecular targets overlap with RB-related pathological programs remains unclear. In this study, we integrated network toxicology, transcriptomic mining, machine learning, molecular docking, and *in vitro* expression validation to construct a candidate TCDD-RB molecular network.

Computational toxicology analysis clarified the structural basis of TCDD as a highly toxic compound at the molecular level. Its molecular features, including high lipophilicity, a rigid planar structure, and low polarity, were highly consistent with the predicted carcinogenicity, genotoxicity, environmental persistence, eye irritation, and multi-organ toxicity. The prediction showed that TCDD had a high probability of eye irritation (0.806), which was consistent with its strong membrane permeability and tissue accumulation potential determined by its physicochemical properties. This suggests that ocular tissues, especially the lipid-rich and highly vascularized retina, may be potential target tissues after systemic TCDD exposure. This potential accumulation effect may affect key biological processes, such as cell cycle regulation, angiogenic signaling, neurotransmission, and intracellular homeostasis, thereby disturbing gene networks related to retinal function and tumorigenesis. These findings provide a rationale for exploring potential molecular links between TCDD-associated toxicological features and RB-related alterations.

Integration of 174 predicted TCDD targets with 4,711 RB differentially expressed genes identified 18 overlapping targets that were enriched in cell-cycle-related pathways, consistent with the proliferative characteristics of RB as a pediatric retinal tumor. These findings indicate potential convergence between predicted TCDD-associated targets and RB-associated transcriptional alterations, but they do not demonstrate that TCDD directly regulates these pathways in RB. WGCNA further identified gene co-expression modules closely associated with RB. Among them, the MEblue module showed the strongest correlation with disease status (*r* = −0.70), indicating its statistical association with RB rather than a TCDD-induced regulatory effect.

Based on systematic screening using eight machine learning algorithms, we identified five hub genes: CDK1, CA14, PIP5K1B, GRIA2, and KDR. These genes showed distinct expression patterns in RB, with CDK1 significantly upregulated and the other four genes downregulated. This pattern suggests that the TCDD-related molecular network may be associated with abnormal cell cycle activation and impaired homeostasis in RB. CDK1 showed excellent discrimination in this dataset (AUC = 0.971), suggesting its potential as a candidate predictive marker.

SHAP analysis improved model interpretability by quantifying the contribution of individual genes to RB prediction. CDK1 showed a positive and feature-dependent contribution to the prediction output, supporting its prioritization as an informative model feature. This analysis moved the machine-learning results beyond simple feature ranking and provided additional model-level evidence for the importance of CDK1. However, SHAP values reflect feature contributions to prediction and should not be interpreted as evidence that CDK1 causally drives RB cell-cycle dysregulation.

Gene interaction network and pathway enrichment analyses identified statistical and functional associations among the five hub genes, with CDK1 occupying a central position in the constructed network. However, these computational associations do not establish direct molecular interactions or cooperative regulatory effects. Correlation analysis showed that the associations between CDK1 and CA14, PIP5K1B, GRIA2, and KDR differed markedly between normal controls and RB tissues, suggesting that gene association patterns may differ between RB and control samples. Although these changes are not sufficient to establish direct molecular causality, they support CDK1 as a candidate hub associated with RB-related molecular alterations.

Among the five hub genes, differential expression analysis showed that CDK1 was the only gene significantly upregulated in RB tissues. GSEA and GSVA based on transcriptomic data showed that samples with high CDK1 expression were significantly enriched in proliferation-related pathways, such as cell cycle, DNA replication, and mismatch repair. This suggests that CDK1 may be closely related to abnormal cell cycle activation in RB by participating in G2/M transition and mitotic progression. In contrast, samples with low CDK1 expression were more enriched in microenvironment-related pathways, such as complement and coagulation cascades, suggesting possible pathway stratification between proliferative status and immune/microenvironment regulation in RB.

Unlike CDK1, CA14, PIP5K1B, GRIA2, and KDR were generally downregulated in RB tissues. Single-gene GSEA and GSVA showed that these downregulated candidate genes were associated with pathways related to the cell cycle, DNA replication and repair, metabolic regulation, neural signaling, and the immune microenvironment. CA14 encodes a carbonic anhydrase family member involved in acid–base balance and pH homeostasis. Its reduced expression may therefore reflect altered metabolic or ionic homeostasis in RB, although its direct role in RB has not been functionally established. PIP5K1B participates in phosphoinositide metabolism and membrane-associated signaling. Its downregulation may therefore be associated with altered lipid-signaling homeostasis rather than an established tumor-suppressive function. GRIA2 encodes an AMPA-type glutamate receptor subunit involved in neural signaling. Its reduced expression may reflect alterations in retinal neuronal signaling programs, which may be relevant to the developmental retinal origin of RB. High KDR expression was enriched in immune-related pathways, including antigen presentation, allograft rejection, and complement and coagulation cascades, whereas low KDR expression was associated with cell-cycle, DNA-replication, and DNA-repair pathways. KDR, also known as VEGFR2, is a major receptor involved in VEGF-mediated angiogenic signaling, and VEGF–VEGFR2 signaling has been implicated in RB angiogenesis and tumor vascularization. However, because KDR expression in bulk transcriptomic data may also be influenced by endothelial-cell abundance and tumor microenvironment composition, its role in RB should be interpreted cautiously. Together, these findings suggest that CA14, PIP5K1B, GRIA2, and KDR may be associated with metabolic, lipid-signaling, neural, angiogenic, and immune-microenvironmental features in RB, but their direct biological functions require further validation.

Together, these findings support a candidate multi-gene framework involving proliferation-related, metabolic, neural-signaling, DNA-repair, angiogenic, and microenvironment-associated processes in RB. However, the coordinated biological roles of these genes and their relationship to TCDD exposure remain to be experimentally established (Griffith et al., 2026).

The pan-cancer analysis was included to determine whether the expression and prognostic patterns of the RB-associated hub genes reflected broader cancer-associated features or were predominantly tumor-context dependent. The recurrent upregulation of CDK1 and its association with poorer overall survival in several cancer types were consistent with its upregulation in RB and provided external contextual support for its prioritization as a proliferation-related hub within the TCDD–RB network. In contrast, CA14, PIP5K1B, GRIA2, and KDR exhibited more heterogeneous expression, prognostic, immune-related, MSI, and TMB associations across cancer types, indicating greater tumor-context dependence. Thus, the pan-cancer findings support the central hypothesis by distinguishing the relatively consistent cancer-associated pattern of CDK1 from the more context-dependent patterns of the other hub genes. However, these analyses provide supportive contextual evidence and do not constitute direct mechanistic evidence for RB or TCDD-mediated regulation.

Molecular docking was used as an exploratory structural prediction approach to evaluate the potential spatial compatibility between TCDD and the five hub proteins. The predicted docking energies between TCDD and the five proteins ranged from −8.3 to −6.8 kcal/mol, with CDK1 showing the lowest predicted docking energy (−8.3 kcal/mol), followed by CA14 (−7.4 kcal/mol), KDR (−6.9 kcal/mol), GRIA2 (−6.8 kcal/mol), and PIP5K1B (−6.8 kcal/mol). These results indicate favorable predicted structural compatibility and plausible docking poses between TCDD and the five hub proteins under the computational docking conditions. However, docking scores and predicted poses should be interpreted as relative structural predictions rather than quantitative evidence of binding affinity, biological activity, or functional interactions. Because experimentally validated TCDD-binding sites were unavailable and the docking search spaces were computationally defined, nonphysiological docking poses and false-positive predictions cannot be excluded. Among the five hub genes, CDK1 was the only one significantly upregulated in RB tissues and showed the most favorable predicted docking energy with TCDD. Because the predicted docking poses were not validated by experimental binding or target-engagement assays, the docking findings should be interpreted as computational prioritization evidence for CDK1 rather than confirmation that TCDD directly binds to or functionally modulates CDK1 in RB cells. Previous studies have confirmed that TCDD is a classical ligand of AHR and that many of its toxic and immunomodulatory effects are mediated through AHR signaling (Deb et al., 2026; Karchner et al., 2026). Thus, the potential relationship between TCDD and CDK1 may also involve indirect AHR-related regulatory mechanisms, which require direct experimental confirmation.

qRT-PCR was used to detect the mRNA expression levels of the five hub genes in ARPE-19 cells, which were used as a non-tumor retinal pigment epithelial reference, and RB cell lines, including Y79 and WERI-RB-1. The results showed that CDK1 was significantly upregulated in RB cells, whereas CA14, PIP5K1B, GRIA2, and KDR were significantly downregulated, directionally consistent with the expression patterns observed in the clinical-sample-based GEO transcriptomic analyses. We successfully screened an effective CDK1 knockdown sequence (sh2) and constructed CDK1 overexpression and knockdown cell models. qRT-PCR and Western blotting confirmed that CDK1 mRNA and protein levels were increased in the overexpression group and decreased in the knockdown group. These cell-line experiments provided secondary, supportive expression-level evidence and verified the efficiency of CDK1 manipulation; they were not intended as independent confirmation of RB-specific differential expression.

However, these expression-level experiments did not establish the functional consequences of altered CDK1 expression in RB cells or a causal relationship between TCDD exposure and CDK1 regulation. Therefore, the proposed TCDD-CDK1 relationship should be interpreted as a hypothesis-generating inference derived from toxicological target prediction, transcriptomic overlap, molecular docking, and supportive expression-level evidence, rather than as direct evidence that TCDD regulates CDK1 or drives RB-related phenotypes through CDK1.

Recent evidence also provides external support for our findings. That study found that CDK1 and CDK2 were highly expressed in RB tissues and cell lines. Pharmacological inhibition of CDK1/2 activity induced G2/M phase arrest, DNA damage, and apoptosis, thereby suppressing RB cell proliferation and tumor growth. These findings further support the potential biological role of CDK1 in RB. Unlike that study, our work identified five hub genes by intersecting TCDD-related toxicological targets with RB-related genes and further validated CDK1 expression changes by qRT-PCR and Western blot. These results are consistent with previous evidence indicating that CDK1 is involved in RB cell-cycle regulation and support CDK1 as a candidate molecule for further investigation within the proposed TCDD-RB network. However, they do not establish that TCDD directly regulates CDK1 or that CDK1 is currently a validated therapeutic target in RB (Yang et al., 2026).

Nevertheless, the biological role of CDK1 may vary across ocular tumor types and regulatory contexts. In RB, Yang et al. showed that pharmacological inhibition of CDK1/2 and combined CDK1/2 knockdown reduced cell viability and induced G2/M arrest and apoptosis, supporting the involvement of the CDK1/2 axis in RB cell proliferation (Yang et al., 2026). However, Li et al. reported a different pattern. Suppression of the TRPM2-AS/miR-497/WEE1 axis inhibited RB cell proliferation, migration, and invasion and increased apoptosis, while total CDK1 expression increased and inhibitory CDK1 Tyr15 phosphorylation decreased (Li et al., 2021). Because CDK1 was not specifically targeted in that study, these findings do not demonstrate a tumor-suppressive role for CDK1. Instead, they indicate that CDK1-related effects may be influenced by phosphorylation status, WEE1-mediated checkpoint regulation, and cellular context. In uveal melanoma, the multi-CDK inhibitor P1446A-05 also inhibited tumor-cell growth and induced G2/M arrest and apoptosis, although the specific contribution of CDK1 could not be separated from that of other CDKs (Eliades et al., 2016). Therefore, our findings support a proliferation-related role for CDK1 in the TCDD-associated RB network but do not establish that CDK1 has a uniform oncogenic function across all ocular tumors.

Based on these findings, we proposed a candidate network model in which TCDD-associated molecular perturbations may be related to RB development. In this model, CDK1-related cell-cycle and proliferative signaling, together with biological processes associated with CA14, PIP5K1B, GRIA2, and KDR, may represent candidate components of the proposed TCDD-RB network. These predicted multi-target associations provide a hypothesis for investigating potential molecular links between environmental pollutant exposure and RB-related alterations. This interpretation is broadly consistent with previous studies suggesting that environmental pollutants may be associated with tumor-related biological processes (Niu et al., 2025). For example, Chen et al. reported that per- and polyfluoroalkyl substances, including PFOS and PFOA, may disrupt hormone balance and induce neurological and immune toxicity in children, potentially contributing to an increased risk of RB (Chen et al., 2024). Together, these observations support further investigation of environmental exposures as potential contributors to RB-related molecular alterations. To avoid overstating causal inference, this model is presented as a hypothesis-generating candidate network rather than a validated molecular mechanism, and a schematic summary is provided in Supplementary Figure S15.

Overall, this study integrates computational toxicology, transcriptomic analysis, machine learning, molecular docking, and expression-level validation to prioritize candidate molecules for subsequent direct TCDD exposure experiments and functional studies.

However, this study has several limitations. First, ARPE-19 cells were used as a non-tumor retinal pigment epithelial reference, but they are not lineage-matched controls for RB, which is a tumor of neural retinal origin. Consequently, some expression differences between ARPE-19 and RB cell lines may reflect lineage-specific expression programs rather than exclusively RB-associated alterations. Accordingly, the primary differential expression evidence was derived from clinical-sample-based GEO transcriptomic analyses, whereas the qRT-PCR comparisons using ARPE-19 cells should be regarded as secondary supportive evidence. Although qRT-PCR supported the observed expression patterns of the hub genes, and qRT-PCR and Western blotting confirmed the efficiency of CDK1 overexpression and knockdown, these experiments provided expression-level validation only and did not assess the effects of CDK1 manipulation on RB cell proliferation, apoptosis, cell-cycle progression, migration, or invasion. These experiments were intended as preliminary molecular validation, whereas functional phenotyping was not included in the current exploratory study design. Second, direct TCDD exposure experiments were not performed in RB cells. Neither AHR activation nor downstream AHR-responsive signaling was evaluated, and AHR inhibition or knockdown was not performed. Therefore, this study cannot determine whether TCDD directly affects CDK1 expression or activity, AHR activation, downstream AHR signaling, or RB-related cellular phenotypes. The proposed TCDD-CDK1 association remains inferential and should not be interpreted as evidence that TCDD directly regulates CDK1 or promotes RB-related phenotypes through CDK1. Third, although molecular docking predicted a plausible docking pose and favorable docking energy between TCDD and CDK1, docking alone cannot establish direct physical binding or functional interaction. Because experimental binding or target-engagement assays were not performed, the docking results cannot confirm direct TCDD-CDK1 binding, cellular target engagement, binding affinity, or functional consequences. Fourth, this study lacked validation in animal models and clinical cohorts with individual-level environmental exposure data. Therefore, the relationships among TCDD exposure or accumulation levels, CDK1 expression, tumor characteristics, and clinical outcomes require further validation.

Future studies should combine direct TCDD exposure experiments with AHR pathway manipulation, CDK1 loss-of-function and rescue assays, analyses of CDK1 expression, phosphorylation, and kinase activity, animal models, and exposure-informed clinical cohorts. Complementary biochemical and biophysical assays, such as surface plasmon resonance, isothermal titration calorimetry, microscale thermophoresis, cellular thermal shift assays, CDK1 kinase activity assays, and substrate phosphorylation analyses, are required to determine whether the predicted structural compatibility corresponds to direct TCDD-CDK1 binding and whether any experimentally confirmed binding has functional consequences. These studies would help clarify whether TCDD-related effects in RB involve an AHR-CDK1-associated pathway. In conclusion, this study integrated computational toxicology, transcriptomic analysis, machine learning, molecular docking, and expression validation to identify a candidate TCDD-RB molecular network and prioritize CDK1 as a hub associated with cell-cycle-related alterations in RB. These findings provide a hypothesis-generating basis for subsequent functional and mechanistic studies of the potential molecular links between environmental pollutants and RB.

## 5 Conclusion

In conclusion, this study integrated network toxicology, RB transcriptomic datasets, machine learning, molecular docking, and *in vitro* expression validation to identify candidate TCDD-associated molecular targets and pathways in RB. CDK1 emerged as an RB-upregulated candidate hub gene associated with cell-cycle-related pathways and showed the most favorable predicted docking energy with TCDD among the five hub proteins. The low expression of CA14, PIP5K1B, GRIA2, and KDR may be related to alterations in metabolic homeostasis, neural signal transduction, angiogenic signaling, and immune microenvironment regulation. *In vitro* validation further supported the dysregulated expression patterns of the hub genes and confirmed the efficiency of CDK1 overexpression and knockdown in RB cells. These findings provide an exploratory and hypothesis-generating framework suggesting that CDK1-related cell-cycle dysregulation may represent a candidate molecular link between predicted TCDD-associated toxicological networks and RB transcriptomic alterations. However, this study does not establish a definitive molecular mechanism or a causal role of TCDD in RB development. Therefore, the identified targets should be interpreted as computationally prioritized candidates rather than experimentally confirmed mediators of TCDD-induced RB progression. Future studies involving direct TCDD exposure, AHR pathway manipulation, CDK1 loss-of-function and rescue assays, and *in vivo* validation are required to validate the proposed regulatory network and clarify causality.

## Data Availability

The original contributions presented in the study are included in the article/Supplementary Material, further inquiries can be directed to the corresponding authors.

## References

[B1] Banerjee P. Kemmler E. Dunkel M. Preissner R. (2024). ProTox 3.0: a webserver for the prediction of toxicity of chemicals. Nucleic Acids Res. 52W1, W513–W520. 10.1093/nar/gkae303 PMC11223834 38647086

[B2] Bockus A. T. Leung S. S. F. Fraga-Walton B. Baldomero M. P. Hernandez L. Dupper N. J. (2025). Discovery of cell-permeable macrocyclic cyclin A/B RxL inhibitors that demonstrate antitumor activity. J. Med. Chem. 6816, 17030–17045. 10.1021/acs.jmedchem.5c00253 40810865 PMC13138696

[B3] Chen Y. Paul K. C. Walker D. I. Jones D. P. Wang X. Ritz B. R. (2024). Neonatal per- and polyfluoroalkyl substance exposure in relation to retinoblastoma. Environ. Res.240Pt 2, 117435. 10.1016/j.envres.2023.117435 PMC10842486 37866539

[B4] Chen P. Liu X. Yuan D. Zheng L. Du Y. Gong D. (2026). Assessing the toxicological effects of exposure to polyethylene terephthalate on hepatocellular carcinoma: insights from network toxicology, molecular docking, molecular dynamics, and experimental validation. Front. Pharmacol. 17, 1772751. 10.3389/fphar.2026.1772751 PMC13143728 42100321

[B5] Daina A. Michielin O. Zoete V. (2019). SwissTargetPrediction: updated data and new features for efficient prediction of protein targets of small molecules. Nucleic Acids Res. 47W1, W357–W364. 10.1093/nar/gkz382 31106366 PMC6602486

[B6] DebA. MiceliA. KaplanB. L. F. (2026). Indole-3-Carbinol (I3C) suppression of IgG2b-triggered signaling in innate cells involves early suppression of p-Akt. Toxicol. Appl. Pharmacol. 513, 117869. 10.1016/j.taap.2026.117869 42134513

[B7] Eliades P. Miller D. M. Miao B. Kumar R. Taylor M. Buch S. (2016). A novel multi-CDK inhibitor P1446A-05 restricts melanoma growth and produces synergistic effects in combination with MAPK pathway inhibitors. Cancer Biol. Ther. 177, 778–784. 10.1080/15384047.2016.1139267 26810603 PMC4970529

[B8] Faust D. Kletting S. Ueberham E. Dietrich C. (2013). Aryl hydrocarbon receptor-dependent cell cycle arrest in isolated mouse oval cells. Toxicol. Lett. 2231, 73–80. 10.1016/j.toxlet.2013.08.022 24013123

[B9] Griffith B. D. Kadiyala P. McGue J. Sun L. Kumar A. Espinoza C. E. (2026). Aryl hydrocarbon receptor ligands drive pancreatic cancer initiation and progression through protumorigenic T-cell polarization. Cancer Discov. 161, 114–134. 10.1158/2159-8290.Cd-25-0377 40905825 PMC12793834

[B10] Guan J. Lu L. Jiang Y. (2025). NSUN2 contributes to the RB malignant progression and glycolysis by mediating the m5C methylation modification of HKDC1. J. Bioenerg. Biomembr. 574-5, 275–287. 10.1007/s10863-025-10062-1 40423890

[B11] Heck J. E. Park A. S. Qiu J. Cockburn M. Ritz B. (2015). Retinoblastoma and ambient exposure to air toxics in the perinatal period. J. Expo. Sci. Environ. Epidemiol. 252, 182–186. 10.1038/jes.2013.84 24280682 PMC4059784

[B12] Huang G. Elferink C. J. (2005). Multiple mechanisms are involved in Ah receptor-mediated cell cycle arrest. Mol. Pharmacol. 671, 88–96. 10.1124/mol.104.002410 15492120

[B13] Kanehisa M. Sato Y. Kawashima M. (2022). KEGG mapping tools for uncovering hidden features in biological data. Protein Sci. 311, 47–53. 10.1002/pro.4172 PMC8740838 34423492

[B14] Kang Z. Liu G. (2026). Exploring multiple biomarkers and constructing ferroptosis-associated competing endogenous RNA networks as dual targets in retinoblastoma. Oncol. Lett. 316, 226. 10.3892/ol.2026.15582 PMC13100569 42027641

[B15] Karchner S. I. Aluru N. Franks D. G. Mandl C. A. Goldstone J. V. Burke T. (2026). Using fish models to understand the role of aryl hydrocarbon receptor (AHR)-interacting protein (AIP) in controlling sensitivity and resistance to dioxin-like compounds *in vivo* . Toxicol. Sci. 2093, kfag033. 10.1093/toxsci/kfag033 41840760 PMC13035081

[B16] Kim S. Chen J. Cheng T. Gindulyte A. He J. He S. (2023). PubChem 2023 update. Nucleic Acids Res. 51D1, D1373–D1380. 10.1093/nar/gkac956 36305812 PMC9825602

[B17] Kim C. Kim J. Shin J. E. Lim S. H. Kim K. M. Kim D. G. (2026). Prognostic implications of tissue-based homologous recombination deficiency in metastatic gastric cancer treated with immune checkpoint inhibitor plus chemotherapy. Gastric Cancer 29, 816–826. 10.1007/s10120-026-01755-6 42171983

[B18] Kumari A. Singh S. P. Kumar P. Kondaveeti S. B. Garg V. K. Kaur R. (2025). A comprehensive review of the epidemiology, pathophysiology, risk factors, and treatment strategies for retinoblastoma. Diseases 139, 307. 10.3390/diseases13090307 41002743 PMC12469102

[B19] Leek J. T. Johnson W. E. Parker H. S. Jaffe A. E. Storey J. D. (2012). The sva package for removing batch effects and other unwanted variation in high-throughput experiments. Bioinformatics 286, 882–883. 10.1093/bioinformatics/bts034 22257669 PMC3307112

[B20] Li A. Yang J. Zhang T. Li L. Li M. (2021). Long noncoding RNA TRPM2-AS promotes the growth, migration, and invasion of retinoblastoma *via* miR-497/WEE1 axis. Front. Pharmacol. 12, 592822. 10.3389/fphar.2021.592822 33986660 PMC8112210

[B21] Li R. Ren C. He L. Wang S. Guo G. (2026). Machine learning-integrated network toxicology uncovers glioma targets of DEHP. Front. Toxicol. 8, 1771011. 10.3389/ftox.2026.1771011 42183337 PMC13193683

[B22] Liu H. Wan L. Fang Q. (2026). A bioinformatics and experimental approach identifies glycoprotein-based diagnostic and prognostic biomarkers for colon adenocarcinoma. Sci. Rep. 16, 22605. 10.1038/s41598-026-52427-5 42151258 PMC13381531

[B23] Liu H. Jiang J. Tan Y. Yang M. Yang H. Li C. (2025). Unraveling diethyl phthalate-induced prostate carcinogenesis: core targets revealed by integrated network toxicology, machine learning, and structural validation. Hum. Genomics 191, 149. 10.1186/s40246-025-00863-1 41444679 PMC12729194

[B24] Liu X. Jiao S. F. Liu C. Y. Luo R. Liu H. Chai Y. (2025). Denticleless homolog-mediated ubiquitin-proteasome degradation of forkhead box O1 contributes to development of carboplatin resistance in retinoblastoma. Anticancer Drugs 368, 648–663. 10.1097/cad.0000000000001738 40455648

[B25] Louzon M. Chiquet C. Kamel M. Aleya L. Blanc H. Blanc A. (2026). Ophthalmotoxicology of polyhalogenated compounds. Toxicology 520, 154337. 10.1016/j.tox.2025.154337 41240996

[B26] Mahmoudi A. Hajihasani M. M. Majeed M. Jamialahmadi T. Sahebkar A. (2024). Effect of Calebin-A on critical genes related to NAFLD: a protein-protein interaction network and molecular docking Study. Curr. Genomics 252, 120–139. 10.2174/0113892029280454240214072212 PMC11092913 38751599

[B27] Mendez D. Gaulton A. Bento A. P. Chambers J. De Veij M. Félix E. (2019). ChEMBL: towards direct deposition of bioassay data. Nucleic Acids Res. 47D1, D930–D940. 10.1093/nar/gky1075 30398643 PMC6323927

[B28] Niu Y. Lee H. J. Chen Z. Kim H. Kim K. (2025). Effects of the carcinogen TCDD on δ-catenin stability and tumorigenic potential in prostate cancer cells. Am. J. Cancer Res. 154, 1939–1954. 10.62347/wrbd9281 PMC12070107 40371154

[B29] Pitarch B. Chagoyen M. Ranea J. A. G. Pazos F. (2025). A review on Gene Ontology evaluations. Database (Oxford) 2025, baaf058. 10.1093/database/baaf058 PMC12462381 40996702

[B30] Qi X. Wang S. Fang C. Jia J. Lin L. Yuan T. (2025). Machine learning and SHAP value interpretation for predicting comorbidity of cardiovascular disease and cancer with dietary antioxidants. Redox Biol. 79, 103470. 10.1016/j.redox.2024.103470 PMC11729017 39700695

[B31] Ravindranath P. A. Forli S. Goodsell D. S. Olson A. J. Sanner M. F. (2015). AutoDockFR: advances in protein-ligand docking with explicitly specified binding site flexibility. PLoS Comput. Biol. 1112, e1004586. 10.1371/journal.pcbi.1004586 26629955 PMC4667975

[B32] Rosignoli S. Paiardini A. (2022). Boosting the full potential of PyMOL with structural biology plugins. Biomolecules 1212, 1764. 10.3390/biom12121764 PMC9775141 36551192

[B33] Si S. Liu J. Li Z. Wang P. Ma Y. (2026). Elucidating the potential mechanism of short-chain chlorinated paraffins in breast cancer via computational prediction integrating network toxicology and molecular docking. Sci. Rep. 161, 15792. 10.1038/s41598-026-44845-2 41927749 PMC13195138

[B34] Subramanian A. Tamayo P. Mootha V. K. Mukherjee S. Ebert B. L. Gillette M. A. (2005). Gene set enrichment analysis: a knowledge-based approach for interpreting genome-wide expression profiles. Proc. Natl. Acad. Sci. U. S. A. 10243, 15545–15550. 10.1073/pnas.0506580102 16199517 PMC1239896

[B35] Szklarczyk D. Kirsch R. Koutrouli M. Nastou K. Mehryary F. Hachilif R. (2023). The STRING database in 2023: protein-protein association networks and functional enrichment analyses for any sequenced genome of interest. Nucleic Acids Res. 51D1, D638–D646. 10.1093/nar/gkac1000 36370105 PMC9825434

[B36] Takeuchi A. Takeuchi M. Oikawa K. Sonoda K. H. Usui Y. Okunuki Y. (2009). Effects of dioxin on vascular endothelial growth factor (VEGF) production in the retina associated with choroidal neovascularization. Invest Ophthalmol. Vis. Sci. 507, 3410–3416. 10.1167/iovs.08-2299 19182260

[B37] Thorsson V. Gibbs D. L. Brown S. D. Wolf D. Bortone D. S. Ou Yang T. H. (2018). The immune landscape of cancer. Immunity 484, 812–830.e14. 10.1016/j.immuni.2018.03.023 29628290 PMC5982584

[B38] Trott O. Olson A. J. (2010). AutoDock vina: improving the speed and accuracy of docking with a new scoring function, efficient optimization, and multithreading. J. Comput. Chem. 312, 455–461. 10.1002/jcc.21334 19499576 PMC3041641

[B39] Vaithianathan A. M. Zanazzi G. (2026). Retinoblastoma and its tumor microenvironment. Curr. Oncol. 335, 264. 10.3390/curroncol33050264 42187581 PMC13205838

[B40] Wang W. Hwang S. Park D. Park Y. D. (2024). The features of shared genes among transcriptomes probed in atopic dermatitis, psoriasis, and inflammatory acne: S100A9 selection as the target gene. Protein Pept. Lett. 315, 356–374. 10.2174/0109298665290166240426072642 38766834

[B41] Wang L. Chen J. Shen Y. Hooi G. L. M. Wu S. Xu F. (2025). Incidence, mortality, and global burden of retinoblastoma in 204 countries worldwide from 1990 to 2021: data and systematic analysis from the global burden of disease study 2021. Neoplasia 60, 101107. 10.1016/j.neo.2024.101107 39724751 PMC11730568

[B42] Wang G. Feng Q. Zhu G. (2026). Development of a prognostic model for thyroid cancer based on mitochondrial metabolism-related genes and immune profiling. World J. Surg. Oncol. 241, 67. 10.1186/s12957-025-04169-3 41495747 PMC12871013

[B43] Wu D. Gao M. Zhou B. Zhu C. Luo Y. Xia F. (2025). FAAP100:A biomarker based on pan-cancer analysis, promotes the progression of lung adenocarcinoma. Cell Signal 134, 111950. 10.1016/j.cellsig.2025.111950 40541815

[B44] Xiong G. Wu Z. Yi J. Fu L. Yang Z. Hsieh C. (2021). ADMETlab 2.0: an integrated online platform for accurate and comprehensive predictions of ADMET properties. Nucleic Acids Res. 49W1, W5–w14. 10.1093/nar/gkab255 33893803 PMC8262709

[B45] Xu Y. Ning L. Wang R. Ma H. Zhao H. Si Y. (2026). Integrative metabolomics and machine learning reveal the toxic mechanisms of elaidic acid in polycystic ovary syndrome. Reprod. Toxicol. 141, 109200. 10.1016/j.reprotox.2026.109200 41747841

[B46] Yang H. Xiao Q. Shen Y. Yao Y. Yin X. Sang Y. (2026). Abemaciclib inhibits retinoblastoma tumor growth by targeting CDK1/2. Invest Ophthalmol. Vis. Sci. 675, 72. 10.1167/iovs.67.5.72 42212883 PMC13225307

[B47] Zheng R. Lu J. Deng M. Lyu J. Han J. Chen J. (2025). Baricitinib in chronic kidney disease: an exploratory analysis integrating network toxicology, molecular docking and pharmacovigilance. Front. Med. (Lausanne) 12, 1717739. 10.3389/fmed.2025.1717739 PMC12827112 41585231

