# Supplementary material for: Computational prioritization of candidate TCDD-associated targets in retinoblastoma using network toxicology and transcriptomic analysis
Source: Front Genet. 2026 Sep 9;17:1911449. doi: 10.3389/fgene.2026.1911449 (PMC13597058; doi:10.3389/fgene.2026.1911449)
Supplement: Supplementary file 2 [file Supplementaryfile1.docx]

Supplementary Material

# Supplementary Methods

## Supplementary Methods S1. Molecular docking details and protocol validation

The molecular structure of TCDD was obtained from PubChem and saved in SDF format. The experimentally determined human protein structures of CDK1 (PDB ID: 4Y72), CA14 (PDB ID: 5CJF), GRIA2 (PDB ID: 2WJW), and KDR (PDB ID: 1YWN) were obtained from the RCSB PDB database. These structures were selected based on their human origin, structural quality, coverage of the relevant protein domains, and suitability for molecular docking. Because a suitable experimentally determined structure was unavailable for PIP5K1B, the AlphaFold-predicted human PIP5K1B structure corresponding to UniProt accession O14986 was used. Detailed structural information and selection rationales are provided in Supplementary Table S4.

Water molecules and original ligands were removed using PyMOL, while the co-crystallized ligands required for protocol validation were retained separately. The processed protein structures and TCDD were converted into PDBQT format using AutoDockTools, and molecular docking was then performed using AutoDock Vina. Because experimentally validated TCDD-binding sites were unavailable, broad target-specific search spaces were defined using the GetBox Plugin. Molecular docking was performed using AutoDock Vina, with an exhaustiveness value of 32 and up to 10 binding modes. The corresponding grid-box parameters are provided in Supplementary Table S3.

For protocol validation, the co-crystallized ligands LZ9, 520, and LIF were extracted from the CDK1, CA14, and KDR crystal structures, respectively, and redocked into their native ligand-binding pockets using the same receptor and ligand preparation procedures and docking settings. The top-ranked redocked poses were superimposed onto the corresponding crystallographic ligand poses, and heavy-atom RMSD values were calculated using PyMOL. An RMSD below 2.0 Å was considered to indicate acceptable reproduction of the crystallographic binding pose. Redocking validation was not performed for GRIA2 or PIP5K1B because suitable co-crystallized small-molecule ligands were unavailable.

### **Supplementary Results S1. Detailed Pan-Cancer Expression and Prognostic Analyses of the Five Hub Genes**

S1.1 Pan-Cancer Expression and Correlation Patterns

The expression patterns of CDK1, CA14, GRIA2, KDR, and PIP5K1B were evaluated across 33 TCGA cancer types. CDK1 showed relatively high expression across multiple tumour types and was significantly upregulated in breast invasive carcinoma (BRCA), cervical squamous cell carcinoma and endocervical adenocarcinoma (CESC), cholangiocarcinoma (CHOL), colon adenocarcinoma (COAD), and glioblastoma multiforme (GBM). In contrast, the other four genes showed more heterogeneous expression patterns, with downregulation observed in several cancer types. CA14 was downregulated in prostate adenocarcinoma (PRAD), rectum adenocarcinoma (READ), and stomach adenocarcinoma (STAD). GRIA2 was downregulated in GBM, kidney chromophobe (KICH), and kidney renal clear cell carcinoma (KIRC). KDR was downregulated in bladder urothelial carcinoma (BLCA) and CHOL. PIP5K1B was downregulated in COAD, BLCA, and CHOL. Inter-gene correlation analysis showed generally weak correlations among the five genes across pan-cancer samples.

S1.2 Kaplan–Meier Survival Analysis

Kaplan–Meier analysis showed that high CDK1 expression was significantly associated with poorer overall survival in brain lower-grade glioma (LGG) and adrenocortical carcinoma (ACC). Higher CA14 expression was associated with better overall survival in KIRC and GBM. Higher GRIA2 expression was associated with better overall survival in LGG and ACC. Higher KDR expression was associated with better overall survival in KIRC and acute myeloid leukaemia (LAML). Higher PIP5K1B expression was associated with better overall survival in mesothelioma (MESO) and KIRC.

S1.3 Univariate Cox Regression Analysis

Univariate Cox regression analysis showed that higher CDK1 expression was associated with increased mortality risk in ACC, KICH, and pheochromocytoma and paraganglioma (PCPG), with all associations reaching statistical significance (*P* < 0.05). The strongest association was observed in ACC, with a hazard ratio of 3.07 (*P* = 2.12 × 10⁻⁸).

Higher CA14 expression was associated with reduced mortality risk in diffuse large B-cell lymphoma (DLBC), READ, and testicular germ cell tumours (TGCT). Higher GRIA2 expression was associated with reduced mortality risk in CHOL, DLBC, and LAML. Higher KDR expression was associated with reduced mortality risk in KICH and PCPG. Higher PIP5K1B expression was associated with reduced mortality risk in PCPG and PRAD. All of these associations were statistically significant at *P* < 0.05.

# Supplementary Tables

**Table S1. shRNA target sequences used in this study**

| **Name** | **Target sequence (5′–3′)** |
| --- | --- |
| sh-CDK1-1 | GGATGTGCTTATGCAGGATTC |
| sh-CDK1-2 | GGGTCAGCTCGTTACTCAACT |
| sh-CDK1-3 | GGTCAGTACATGGATTCTTCA |
| sh-NC | TTCTCCGAACGTGTCACGT |

**Table S2. Clinical and sample characteristics of the included GEO datasets**

| **GEO dataset** | **Clinical or sample characteristics** | **Sample size** |
| --- | --- | --- |
| GSE97508 | Non-invasive and invasive RB tissues; adult retinal tissues were used as controls | 6 RB samples and 3 control samples |
| GSE58780 | Primary RB tissues; fetal retinal tissues were used as controls | 63 RB samples and 3 control samples |
| GSE111168 | RB tissues; adjacent non-tumour retinal tissues were used as controls | 3 RB samples and 3 control samples |
| GSE87042 | RB tissues; developing human retinal tissues were used as controls | 5 RB samples and 8 control samples |
| GSE214685 | Y79 RB cells; ARPE-19 retinal pigment epithelial cells were used as controls | 10 RB samples and 4 control samples |

Note: Potential heterogeneity among the datasets may arise from differences in sample source and control type, including adult, fetal, para-tumour, and developing retinal tissues, as well as cell-line-derived samples.

**Table S3. Grid-box parameters used for TCDD–protein docking**

| **Target** | **Centre x(Å)** | **Centre y(Å)** | **Centre z(Å)** | **Size x(Å)** | **Size y(Å)** | **Size z(Å)** |
| --- | --- | --- | --- | --- | --- | --- |
| CA14 | 69.6 | 34.7 | 12.5 | 62.9 | 56.9 | 57.9 |
| GRIA2 | −24.1 | −33.7 | −18.7 | 60.3 | 76.0 | 61.4 |
| KDR | 9.0 | 38.6 | 21.6 | 69.0 | 57.8 | 72.2 |
| PIP5K1B | −2.1 | 0.6 | 5.3 | 71.5 | 49.9 | 69.3 |
| CDK1 | 19.6 | −62.2 | 185.1 | 55.3 | 72.1 | 72.3 |

**Note: The exhaustiveness value was fixed at 32, and up to 10 binding modes were generated for each target.**

**Table S4. Structural information, selection rationale, and docking-site definition of target proteins**

| **Target** | **Structure ID** | **Source** | **Resolution (Å)** | **Structural characteristics and selection rationale** | **TCDD docking-site definition** |
| --- | --- | --- | --- | --- | --- |
| CDK1 | 4Y72 | RCSB PDB | 2.30 | Experimentally determined human ligand-bound structure with adequate structural quality and relevant domain coverage | GetBox-defined search space because no experimentally validated TCDD-binding site was available |
| CA14 | 5CJF | RCSB PDB | 1.83 | Experimentally determined human ligand-bound structure with adequate structural quality and relevant domain coverage | GetBox-defined search space because no experimentally validated TCDD-binding site was available |
| GRIA2 | 2WJW | RCSB PDB | 1.80 | Experimentally determined human structure covering the available amino-terminal domain | GetBox-defined search space because no experimentally validated TCDD-binding site was available |
| KDR | 1YWN | RCSB PDB | 1.71 | Experimentally determined human ligand-bound kinase-domain structure with adequate structural quality; engineered mutations were present | GetBox-defined search space because no experimentally validated TCDD-binding site was available |
| PIP5K1B | O14986 | AlphaFold Protein Structure Database | Not applicable | Predicted human structure used because a suitable experimentally determined structure was unavailable | GetBox-defined search space because no experimentally validated TCDD-binding site was available |

**Table S5. Validation of the molecular docking protocol by co-crystallized ligand redocking**

| **Target** | **Structure ID** | **Co-crystallized ligand** | **Redocking site** | **Heavy-atom RMSD (Å)** | **Evaluation** |
| --- | --- | --- | --- | --- | --- |
| CDK1 | 4Y72 | LZ9 | Native crystallographic ligand-binding pocket | 0.5999 | Acceptable |
| CA14 | 5CJF | 520 | Native crystallographic ligand-binding pocket | 1.8680 | Acceptable |
| KDR | 1YWN | LIF | Native crystallographic ligand-binding pocket | 0.6989 | Acceptable |

**Note:** The top-ranked redocked pose was compared with the corresponding crystallographic ligand pose. A heavy-atom RMSD below 2.0 Å was considered to indicate acceptable reproduction of the crystallographic binding pose.

## Supplementary Figures

**Supplementary Figure S1.** **Single-gene GSEA of CA14, GRIA2, and PIP5K1B in RB.** RB samples were divided into high- and low-expression groups according to the median expression level of each gene. (A-B) Enriched pathways associated with high and low CA14 expression. (C-D) Enriched pathways associated with high and low GRIA2 expression. (E-F) Enriched pathways associated with high and low PIP5K1B expression.

**Supplementary Figure S2. Validation of the molecular docking protocol by redocking of co-crystallized ligands.** Superposition of the top-ranked redocked ligand pose and the corresponding crystallographic ligand pose for (A) CDK1 (PDB ID: 4Y72, ligand: LZ9, heavy-atom RMSD = 0.5999 Å), (B) CA14 (PDB ID: 5CJF, ligand: 520, heavy-atom RMSD = 1.8680 Å), and (C) KDR (PDB ID: 1YWN, ligand: LIF, heavy-atom RMSD = 0.6989 Å). The enlarged views show the agreement between the redocked and crystallographic ligand poses within their respective native binding pockets. All RMSD values were below the predefined threshold of 2.0 Å.

**Supplementary Figure S3. Associations of CA14, CDK1, and GRIA2 expression with overall survival across cancer types.** Kaplan-Meier curves compare overall survival between the high- and low-expression groups. (A-E) CA14 in ACC, GBM, PCPG, LGG, and SKCM. (F-O) CDK1 in ACC, KIRC, KIRP, LIHC, LUAD, MESO, PAAD, PRAD, SARC, and THYM. (P) GRIA2 in ACC. Red and blue curves indicate the high- and low-expression groups, respectively.

**Supplementary Figure S4.** **Kaplan–Meier survival analysis of GRIA2, KDR, and PIP5K1B in selected cancer types.** (A-E) Overall survival analysis of GRIA2 in KICH, KIRC, LIHC, PAAD, and SKCM. (F-J) Overall survival analysis of KDR in HNSC, LAML, LIHC, LUAD, and UVM. High KDR expression was associated with favourable overall survival in LAML. (K-L) Overall survival analysis of PIP5K1B in DLBC and KIRC. High PIP5K1B expression was associated with favourable overall survival in KIRC. Red curves represent high expression, and blue curves represent low expression.

**Supplementary Figure S5. Associations between CA14 expression and immune cell infiltration across cancer types.** Scatter plots show selected correlations between CA14 expression and CIBERSORT-estimated immune cell infiltration. (A) BRCA. (B) CESC, COAD, and ESCA. (C) ESCA, GBM, and HNSC. (D) HNSC, KIRC, and KIRP. (E) KIRP and LAML. (F) LGG, LUAD, PAAD, and PRAD. (G) TGCT and THCA. (H) THCA. (I) THCA and THYM. (J) UCEC and UCS. The corresponding immune cell populations, correlation coefficients, and *P* values are shown in each plot.

**Supplementary Figure S6. Associations between CDK1 expression and immune cell infiltration across cancer types.** Scatter plots show selected correlations between CDK1 expression and CIBERSORT-estimated immune cell infiltration. (A) ACC, GBM, and KIRP. (B) LAML, LGG, and LUAD. (C) STAD and TGCT. (D) THCA and THYM. (E) BRCA and UCEC. The corresponding immune cell populations, correlation coefficients, and *P* values are shown in each plot.

**Supplementary Figure S7. Associations between GRIA2 expression and immune cell infiltration across cancer types.** (A) Activated dendritic cells, M0 macrophages, M1 macrophages, and M2 macrophages in BRCA. (B) Resting mast cells and activated memory CD4 T cells in BRCA, and naive B cells and resting mast cells in CESC. (C) Resting memory CD4 T cells in CESC, activated NK cells in COAD, and activated and resting memory CD4 T cells in GBM. (D) Neutrophils in KIRC, M2 macrophages and resting mast cells in KIRP, and M0 macrophages in LGG. (E) CD8 T cells in LGG and PRAD, regulatory T cells in SKCM, and resting mast cells in STAD. (F) M0 macrophages, M2 macrophages, and resting memory CD4 T cells in THCA, and resting memory CD4 T cells in UCEC.

**Supplementary Figure S8. Associations between KDR expression and immune cell infiltration across cancer types.** Scatter plots show selected correlations between KDR expression and CIBERSORT-estimated immune cell infiltration. The fitted regression line, 95% confidence interval, correlation coefficient, and P value are shown in each plot. (A) TGCT, THYM, and LUSC. (B) BRCA, LUSC, CESC, and PRAD. (C) COAD, ESCA, and PRAD. (D) HNSC and READ. (E) SKCM, HNSC, and TGCT. (F) TGCT, KIRC, UCEC, and KIRP. (G) KIRP and LAML. (H) LGG, LIHC, and LUAD. The corresponding immune cell populations are labeled in each plot.

**Supplementary Figure S9.** **Associations between PIP5K1B expression and immune cell infiltration across cancer types.** Scatter plots show selected correlations between PIP5K1B expression and CIBERSORT-estimated immune cell infiltration. (A) BLCA, BRCA, and CESC. (B) CESC and ESCA. (C) HNSC and KIRC. (D) KIRC, LIHC, and MESO. (E) READ, SARC, and SKCM. (F) SKCM, STAD, and TGCT. (G) THCA and THYM. (H) THYM and UCEC. The corresponding immune cell populations, correlation coefficients, and *P* values are shown in each plot.

**Supplementary Figure S10.** **Associations between CA14 expression and tumor microenvironment scores across cancer types.** Scatter plots show selected correlations between CA14 expression and ImmuneScore or StromalScore. (A) BLCA and BRCA. (B) CESC and GBM. (C) HNSC and KIRP. (D) LUAD and LUSC. (E) SKCM and THCA. (F) UCEC and PAAD. (G) SARC, TGCT, and PRAD. (H) THYM, LIHC, KIRC, and MESO. The corresponding score type, correlation coefficient, and *P* value are shown in each plot. Cancer abbreviations follow TCGA nomenclature.

**Supplementary Figure S11. Associations between CDK1 expression and tumor microenvironment scores across cancer types.** Scatter plots show selected correlations between CDK1 expression and ImmuneScore or StromalScore. (A) BLCA, BRCA, CESC, and COAD. (B) GBM, HNSC, and KIRC. (C) LIHC, LUSC, PAAD, and SARC. (D) TGCT, STAD, SKCM, and THCA. (E) THCA, THYM, and UCEC. The corresponding score type, correlation coefficient, and *P* value are shown in each plot.

**Supplementary Figure S12.** **Correlation analysis between GRIA2 expression and tumor microenvironment scores across multiple cancer types.** (A) GRIA2 was negatively correlated with both ImmuneScore and StromalScore in BRCA, positively correlated with StromalScore in COAD, and negatively correlated with StromalScore in PCPG. (B) GRIA2 was negatively correlated with both ImmuneScore and StromalScore in GBM and LGG. (C) GRIA2 was negatively correlated with ImmuneScore in TGCT and THYM, and negatively correlated with both ImmuneScore and StromalScore in SARC.

**Supplementary Figure S13. Associations between KDR expression and tumor microenvironment scores across cancer types.** Scatter plots show selected correlations between KDR expression and ImmuneScore or StromalScore. (A) ACC, BLCA, and BRCA. (B) COAD, CESC, and DLBC. (C) ESCA and HNSC. (D) KICH, KIRC, KIRP, and LGG. (E) LIHC, LUAD, and OV. (F) PAAD, PCPG, and SARC. (G) PRAD and READ. (H) UVM, SKCM, and STAD. (I) TGCT, THCA, and UCEC. (J) THYM and UCS. The corresponding score type, correlation coefficient, and *P* value are shown in each plot.

**Supplementary Figure S14. Associations between PIP5K1B expression and tumor microenvironment scores across cancer types.** Scatter plots show selected correlations between PIP5K1B expression and ImmuneScore or StromalScore. (A) BRCA, CHOL, and COAD. (B) HNSC, KIRC, and KIRP. (C) LGG, PAAD, and LUSC. (D) PRAD and SKCM. (E) STAD, TGCT, THCA, and THYM. The corresponding score type, correlation coefficient, and *P* value are shown in each plot.

**Supplementary Figure S15. Proposed hypothesis-generating TCDD-AHR-CDK1-related candidate network in RB.** Integrated toxicological target prediction, RB transcriptomic analysis, machine-learning prioritization, molecular docking, and expression-level validation identified CDK1 as an RB-upregulated candidate hub gene associated with cell-cycle-related pathways. CA14, PIP5K1B, GRIA2, and KDR/VEGFR2 were downregulated and may be associated with pH/metabolic homeostasis, phosphoinositide signaling, retinal neuronal signaling, angiogenic signaling, and immune-microenvironmental features. The TCDD-AHR relationship is based on canonical literature-supported signaling, whereas the proposed AHR-CDK1 and CDK1-related RB associations remain inferential. Dashed arrows indicate hypothetical relationships requiring functional and causal validation.

**Supplementary Figure S16. Full, uncropped western blot membrane images.**
Full, uncropped western blot membrane images corresponding to the CDK1 and β-actin blots shown in Figure 13E.
